# Supplementary material for: Adding evidence of the effects of treatments into relevant Wikipedia pages: a randomised trial
Source: BMJ Open. 2020 Feb 20;10(2):e033655. doi: 10.1136/bmjopen-2019-033655 (PMC7045027; doi:10.1136/bmjopen-2019-033655)
Supplement: Author's manuscript [file bmjopen-2019-033655.draft_revisions.pdf]

BMJ Open is committed to open peer review. As part of this commitment we make the peer review history of every article we publish publicly available.

When an article is published we post the peer reviewers' comments and the authors' responses online. We also post the versions of the paper that were used during peer review. These are the versions that the peer review comments apply to.

The versions of the paper that follow are the versions that were submitted during the peer review process. They are not the versions of record or the final published versions. They should not be cited or distributed as the published version of this manuscript.

BMJ Open is an open access journal and the full, final, typeset and author-corrected version of record of the manuscript is available on our site with no access controls, subscription charges or pay-per-view fees (http://bmjopen.bmj.com).

If you have any questions on BMJ Open's open peer review process please email info.bmjopen@bmj.com

BMJ Open

The effects of Wikipedia referencing: a randomised trial

Journal:	*BMJ Open*
Manuscript ID	bmjopen-2019-033655
Article Type:	Research
Date Submitted by the Author:	16-Aug-2019

Complete List of Authors:	Adams, Clive; University of Nottingham, Division of Psychiatry and Applied Psychology<br>Montgomery, Alan; University of Nottingham, Nottingham Clinical Trials Unit<br>Aburrow, Tony; John Wiley and Sons, Limited<br>Bloomfield, Sophie; East Kent Hospitals University NHS Foundation Trust<br>Briley, Paul; University of Nottingham, Division of Psychiatry and Applied Psychology<br>Carew, Ebun; Nottingham University Hospitals Healthcare NHS Trust<br>Chatterjee-Woolman, Suravi; Sherwood Forest Hospitals NHS Foundation Trust<br>Feddah, Ghalia; Sherwood Forest Hospitals NHS Foundation Trust<br>Friedel, Johannes; University of Aalen<br>Gibbard, Josh; Morecambe Bay Hospitals NHS Trust<br>Haynes, Euan; Gateshead Health NHS Foundation Trust<br>Hussein, Mohsin; University Hospitals of Leicester NHS Trust<br>Jayaram, Mahesh; University of Melbourne, Psychaitry<br>Naylor, Samuel; Sherwood Forest Hospitals NHS Foundation Trust<br>Perry, Luke; Royal Melbourne Hospital<br>Schmidt, Lena; University of Bristol Faculty of Health Sciences<br>Siddique, Umer; Barking Havering and Redbridge Hospitals NHS Trust<br>Tabaksert, Ayla; Northumbria Healthcare NHS Foundation Trust<br>Taylor, Douglas; Wikimedia Foundation Inc<br>Velani, Aarti; Nottingham University Hospitals NHS Trust<br>White, Douglas; Epsom and Saint Helier University Hospitals NHS Trust Epsom Hospital<br>Xia, Jun; Nottingham China Health Institute, The University of Nottingham Ningbo

Keywords:	MEDICAL EDUCATION & TRAINING, Schizophrenia & psychotic disorders < PSYCHIATRY, World Wide Web technology < BIOTECHNOLOGY & BIOINFORMATICS

SCHOLARONE™
Manuscripts

I, the Submitting Author has the right to grant and does grant on behalf of all authors of the Work (as defined in the below author licence), an exclusive licence and/or a non-exclusive licence for contributions from authors who are: i) UK Crown employees; ii) where BMJ has agreed a CC-BY licence shall apply, and/or iii) in accordance with the terms applicable for US Federal Government officers or employees acting as part of their official duties; on a worldwide, perpetual, irrevocable, royalty-free basis to BMJ Publishing Group Ltd ("BMJ") its licensees and where the relevant Journal is co-owned by BMJ to the co-owners of the Journal, to publish the Work in this journal and any other BMJ products and to exploit all rights, as set out in our licence.

The Submitting Author accepts and understands that any supply made under these terms is made by BMJ to the Submitting Author unless you are acting as an employee on behalf of your employer or a postgraduate student of an affiliated institution which is paying any applicable article publishing charge ("APC") for Open Access articles. Where the Submitting Author wishes to make the Work available on an Open Access basis (and intends to pay the relevant APC), the terms of reuse of such Open Access shall be governed by a Creative Commons licence – details of these licences and which Creative Commons licence will apply to this Work are set out in our licence referred to above.

Other than as permitted in any relevant BMJ Author's Self Archiving Policies, I confirm this Work has not been accepted for publication elsewhere, is not being considered for publication elsewhere and does not duplicate material already published. I confirm all authors consent to publication of this Work and authorise the granting of this licence.

For peer review only

Title: The effects of Wikipedia referencing: a randomised trial.

Authors:

Clive E Adams, Alan A Montgomery, Tony Aburrow, Sophie Bloomfield, Paul M Briley, Ebun Carew, Suravi Chatterjee-Woolman, Ghalia Feddah, Johannes Friedel, Josh Gibbard, Euan Haynes, Mohsin Hussein, Mahesh Jayaram, Samuel D Naylor, Luke Perry, Lena Schmidt, Umer Siddique, Ayla S Tabaksert, Doug Taylor, Aarti Velani, Douglas White, Jun Xia

Contact:

Professor Clive E Adams

Chair Mental Health Services Research, Co-ordinating Editor, Cochrane Schizophrenia

Institute of Mental Health

Jubilee Campus

University of Nottingham Innovation Park

Triumph Road

Nottingham

NG7 2TU

Phone: 0115 823 1294

Fax: 0115 823 1294

Email: clive.adams@nottingham.ac.uk

Co-authors:

Alan A Montgomery, Nottingham Clinical Trials Unit, University of Nottingham, Nottingham, UK,
Tony Aburrow, John Wiley Ltd., Chichester, UK,

Sophie Bloomfield, East Kent Hospitals University Foundation Trust, Ashford, UK

Paul M Briley, Institute of Mental Health, University of Nottingham, Nottingham, UK

Ebun Carew, Nottingham University Hospitals Healthcare NHS Trust, Nottingham, UK

For peer review only

Suravi Chatterjee-Woolman, Sherwood Forest Hospitals NHS Foundation Trust, Sutton in Ashfield, UK

Ghalia Feddah, Sherwood Forest Hospitals NHS Foundation Trust, Sutton in Ashfield, UK

Johannes Friedel, University of Aalen, Aalen, Germany

Josh Gibbard, Morecambe Bay Hospitals NHS Trust, Lancaster, UK

Euan Haynes, Gateshead Health, NHS Foundation Trust, Gateshead, UK

Mohsin Hussein, Leicester Evington Centre, Leicester, UK

Mahesh Jayaram, Department of Psychiatry, University of Melbourne, Australia

Samuel D Naylor, Sherwood Forest Hospitals NHS Foundation Trust, Sutton in Ashfield, UK

Luke Perry, Royal Melbourne Hospital, Parkville, Australia

Lena Schmidt, Hochschule Furtwangen University, Furtwangen, Germany; University of Bristol, Bristol, UK

Umer Siddique, Barking, Havering and Redbridge Hospitals NHS Trust, London, UK

Ayla S Tabaksert, Northumbria NHS Foundation Trust, Newcastle upon Tyne, UK

Doug Taylor, Wikimedia UK, Loughborough, UK

Aarti Velani, Nottingham University Hospitals Healthcare NHS Trust, Nottingham, UK

Douglas White, Epsom and St Helier University Hospitals NHS Trust, London, UK

Jun Xia, Nottingham China Health Institute, The University of Nottingham Ningbo, Ningbo, China

Key words

Medical education

Health Informatics

Psychiatry

Randomized controlled trial

Word count 3954

For peer review only

Abstract

Objectives

To evaluate the effects of placement of high-quality evidence within Wikipedia on routinely collected data.

Setting

Wikipedia, Cochrane summary pages and the Cochrane Library.

Design

Randomised trial.

Participants

Up-to-date Cochrane Schizophrenia systematic reviews for which there was a clearly relevant and specific Wikipedia page.

Interventions

Reviews in the intervention group had summary findings tables and hyperlinks to the source text placed in their relevant Wikipedia pages. Wikipedia pages of reviews in the control group were left unchanged.

Main outcome measures

Routinely collected data on access to the full text and summary web-page (after 12 months).

Results

We randomised 70 Cochrane reviews (100% follow up). Six of the 35 Wikipedia pages in the intervention group had the tabular format deleted during the study but all pages continued to report the same data within the text. There was no evidence of effect on either of the co-primary outcomes: full text access adjusted ratio of geometric means 1.30, 95% CI 0.71 to 2.38; page views 1.14, 95% CI 0.6 to 2.13. Results were similar for all other outcomes, with exception of Altmetric score for which there was some evidence of effect (1.36, 95% CI 1.05 to 1.78.

For peer review only

Conclusions

Recording of outcomes necessitated readers of Wikipedia to click out to external sources of information. For every person who sought and clicked the reference on the Wikipedia page to seek more information (the primary outcome), many more were informed by the page alone. Enriching Wikipedia content with summary tables from level 1 evidence is, potentially, a powerful way to improve health literacy. The pursuit of fair balance within Wikipedia health care pages is impressive and its reach unsurpassed. It is possible to test the effects of seeding pages with evidence and this trial should be replicated, expanded and developed.

Trial registration

IRCT2017070330407N2 [Abstract 298 words]

Strengths and limitations of this study

- First randomised trial of evidence placed within Wikipedia pages
- Use of routine data to allow 100% follow up
- Open editing of Wikipedia pages – both intervention and control pages - by the Wikipedia community served to minimise difference between groups.
- Recording of outcome necessitated unusual levels of interest and commitment on the part of the Wikipedia page reader
- Small study in highly specialised area of heath care

For peer review only

Background

Wikipedia is a free-content online encyclopaedia containing articles on a vast range of topics(1). The name itself is a portmanteau of an Hawaiian language word “wiki” meaning quick, and the word encyclopaedia(1). At present there are over 5.7m articles, 46 million pages in the English language(2). Since its creation in 2001, Wikipedia has expanded to attract over 27m registered users(3) with 16 billion pages views per month(4). This made Wikipedia the 5th most popular site on the internet in 2017(5).

Wikipedia is openly-editable. This means that any one of these users can access *and edit* the majority of articles. Wikipedia policy states, however, that all information presented in pages must be “verifiable against a published reliable source”(1). Therefore, all pages aim to contain references for the information they provide. To prevent the risk of pages being devalued with misinformation Wikipedia has various quality control measures. These include; a ‘watchlist’ to notify editors when a page has been edited, a published list of recent changes that editors can access to review, automated computer scripts, page protection on more controversial pages, edit filters on certain pages and blocking any editors who repeatedly damage the value of the page (6). On top of this, Wikipedia has a team of administrators. They are editors who have been given access to additional tools on their account. These include the ability to block/unblock accounts, edit fully protected pages and delete/undelete pages. There are 1,194 administrators on the English language Wikipedia (as of December 2018)(2).

Wikipedia contains many pages relating to healthcare. In 2014 the English language version was estimated to contain 25,000 articles on health-related topics, while across all languages there are 155,000 articles containing 950,000 references(7). These are often accessed via search engine results with one survey suggesting that around 22% of healthcare-related online searches direct to Wikipedia pages(8,9). In 2013 health pages on Wikipedia received 4.8 billion views, making it one of the most used means for accessing health information globally(10). When use of Wikipedia is studied in medical students and doctors, it is clear that it is becoming an increasingly popular resource(11,12). This is, perhaps, enhanced by Wikipedia being entirely free of charge – including data download charges in low and middle income countries. In this context there is criticism that as Wikipedia is openly editable, the information it contains may be unreliable. Some evidence suggests, however, that there is

For peer review only

no difference in accuracy when Wikipedia is compared to other professionally maintained medical databases(13) although opinions differ by subspecialty, depending on the 'target' readership and across time (Table 1).

Table 1: Selection of studies of Wikipedia's value to different readerships by medical subspecialty

Sub-specialty (reference)	Date	Assessing for suitability for….	Conclusion
**10 most costly conditions (14)**	2014	General readership	Most Wikipedia articles representing the 10 most costly medical conditions [….] contain many errors when checked against standard peer-reviewed sources. Caution should be used […..]
**Cancer – general (13)**	2011	Patients	Wiki resource had similar accuracy and depth as the professionally edited database
**Cancer – osteoscarcoma (15)**	2010	Patients	[…] the quality of osteosarcoma-related information found in the English Wikipedia is good but inferior to the patient information provided by the NCI.
**Cardiovascular (16)**	2015	Medical students	Wikipedia entries are not aimed at a medical audience and should not be used as a substitute to recommended medical resources. Course designers and students should be aware that Wikipedia entries on cardiovascular diseases lack accuracy, predominantly due to errors of omission.
**Complementary medicine (17)**	2014	General readership	Patients and health professionals should not rely solely on Wikipedia for information on these herbal supplements when treatment decisions are being made.
**Gastro – hepatology (18)**	2014	Medical students	…..not good source of evidence
**Mental health**	2012	General	The quality of information on depression and schizophrenia on Wikipedia is generally as good as, or

**(19)**		readership	better than, that provided by centrally controlled websites, Encyclopaedia Britannica and a psychiatry textbook.
**Nephrology (20)**	2013	Patients	Fairly reliable medical resource
**Orthognathic surgery (21)**	2012	Patients	Maximum […] score[ings in comparison to other online sources] were Wikipedia
**Pharmacology (22)**	2017	Doctors	Wikipedia lacks the accuracy and completeness of standard clinical references and should not be a routine part of clinical decision making.
**Pharmacology (23)**	2014	Medical students	… Wikipedia is an accurate and comprehensive source of drug-related information for undergraduate medical education.
**Pharmacology (24)**	2008	Patients	Wikipedia has a more narrow scope, is less complete, and has more errors of omission than the comparator database. Wikipedia may be a useful point of engagement for consumers, but is not authoritative and should only be a supplemental source of drug information.
**Respiratory medicine (25)**	2015	Medical students	Most articles had knowledge deficiencies, were not accurate, and were not suitable for medical students as learning resources.

The Cochrane Collaboration is a non-for profit NGO producing, and maintaining systematic reviews of health care. A systematic review "attempts to collate all empirical evidence that fits pre-specified eligibility criteria in order to answer a specific research question. It uses explicit, systematic methods that are selected with a view to minimizing bias, thus providing more reliable findings from which conclusions can be drawn and decisions made."(26) Cochrane Reviews are in the *Cochrane Database of Systematic Reviews*; one of the databases included in the *Cochrane Library (*published by John Wiley Ltd.). Full access to the *Cochrane Library* is available to several countries where a national provision has been purchased and freely available to over 120 low and middle-income nations. It is also sold via a subscription model to institutional and individual users.(27). An additional universally accessible 'entry point' to each review is the plain language summary (PLS) on the Cochrane

For peer review only

Library. This award-winning section aims to make the information more accessible to people without specialist knowledge(28) and is accompanied by a more traditional academic abstract of the review and a hyperlink to the full publication. The Collaboration is made up of subgroups and Cochrane Schizophrenia produces and updates high quality systematic reviews and meta-analyses relevant to people with schizophrenia and related psychotic conditions.

In 2004 a group called WikiProject Medicine was started with the aim of creating and managing medical articles on Wikipedia. This group allows discussion and collaboration on these articles to improve the quality of the information presented(6). In 2014 a formalised partnership between Wikipedia and Cochrane was created, aiming to “transform the quality and content of health evidence available online”(29). This involves incorporating Cochrane’s evidence into Wikipedia articles and improving the information’s accuracy and reliability. Whilst increasing accessibility of highest grade maintained health care information seems a laudable aim objective quantification of the effects of this effort has not been undertaken.

Aims

To evaluate the effects of enriching Wikipedia content with summary tables from level 1 evidence on the effects of care.

Methods

In preliminary work we tested stability of target pages in Wikipedia. Adding an evidence-table to four Wikipedia pages (one old little-used drug, one old widely-used drug, one expensive new drug, and one important talking therapy) found that all remained stable over a 12 month period(2015). Further work investigated what proportion of the topics of Cochrane Schizophrenia reviews already had a highly specific page in Wikipedia. In 2016 around half of Cochrane Schizophrenia reviews had an obvious ‘landing’ page directly addressing the topic of the review (30). Then in 2016 we held a one day meeting of student volunteers (medicine and students of applied health sciences), trialists and representatives from Wikipedia and John Wiley Ltd., to plan this trial.(31). The study is a two-arm, parallel, open, randomised controlled trial with a 1:1 allocation ratio.

The aim of this study was to evaluate the impact of seeding relevant Wikipedia pages with evidence from high-grade systematic reviews on information-seeking behaviour.

For peer review only

Eligibility

Inclusion criteria

A Cochrane Schizophrenia systematic review published in the Cochrane Library and judged (by CEA) as up-to-date for which there was a clearly relevant and specific Wikipedia page, and in which there is at least one Summary of Findings (SoF) table. These tables, created within the GradePro(32) system, are succinct summaries of the key outcomes of the review.

Exclusion criteria

If the review was out of date with new important evidence not incorporated, had no SoF table and did not have a Wikipedia page which had been judged to have a title specifically relevant to the content of a Cochrane Schizophrenia review(30). For example, a specialist review such as ‘yoga for schizophrenia’ would be out of place on a general Wikipedia page ‘yoga’ and therefore ineligible. However, should there be a highly specific Wikipedia page ‘yoga for schizophrenia’ then this review would have been eligible.

Randomisation

Reviews were stratified according to type of intervention (drug or other) and amount of access activity in the year prior to baseline (low or high, according to median split). The latter used Google Analytics’ ‘pageviews’ statistic regarding Cochrane’s universally accessible individual review pages(33). The reviews were then allocated to the intervention or control arm by one of the co-authors (AAM) using a computer-generated random number sequence. Allocation was conducted using unique code numbers for each review rather than review title, to avoid risk of selection bias.

Interventions

Experimental group

Reviews in the intervention group had a referenced table(s) automatically generated by use of SEED(34). This open access software, especially created for this study, uses the original Cochrane review file and re-writes the Cochrane Summary of Findings tables in plain English and generates hyperlink references (to both full subscription review and the universally accessible web summary page) (Figure 1, (35)). SEED deposits this code in the computer’s memory in seconds. The intervention group’s Wikipedia editor (LS, JF) had only to paste this

For peer review only

code into the Wikipedia page in the relevant sub-section for the table and hyperlink to appear. This was undertaken across the second week of July 2017.

Control intervention

The control group Wikipedia pages did not have a table or reference added – although seven of these pages already had the Cochrane reference employed. This reference was not removed.

Source of data

The routine data on full review access is collected by the Cochrane Library's publisher, Wiley. These data, kindly supplied by the Cochrane Office John Wiley, report full text downloads, and Altmetric scores; a composite weighted measure of the influence of published work online and via social media platforms – in this case composed from monitoring 17 different platforms/news outlets (full list of platforms, and data-by-platform available in Supplementary file) (36). The full review is widely accessible but not universally so. Neither is the full review succinct. Cochrane Summaries web pages are both universally accessible and succinct. They were monitored using the standard (free) service from Google Analytics(33).

Outcomes

All outcomes were measured at 12 months. There were two outcomes of co-primary interest:

1. The number of visits to the free summary page (All page views)
2. The number of full text downloads

We selected these as co-primary outcomes because the design team (REF) felt they represented the best, measurable, most generic measures of 'more interest' in the evidence as presented in the tables. Secondary outcomes were divided into activity on the free to all summary page, and outcomes relating to activity on the Cochrane Library's full review.

Statistical considerations

The sample size for this study is fixed by the number of eligible Cochrane reviews. From preliminary work we had expected to be able to randomise around 100 reviews (30), enabling detection of a between-group standardised difference of 0.57 with 80% power and

For peer review only

5% two-sided alpha. However due to some reviews being too out of date to report on Wikipedia, the actual number available was 70 which permits detection of an effect size of 0.68.

We compared characteristics of the intervention and control arms at baseline using descriptive statistics. For all between-group comparisons, we analysed reviews as randomised regardless of how long the Wikipedia page held the table. We estimated between-group effects using multivariable linear regression models adjusting for baseline activity, presented with 95% confidence intervals and p-values, and with log-transformation of outcomes as required. For such outcomes, results are presented as ratios of geometric means. Data were analysed using Stata version 15.

Results

All 70 eligible Cochrane reviews were randomised, and complete follow up data were available for all reviews (Figure 2).

At baseline Altmetric scores were evenly distributed (Table 2).

Table 2. Baseline altmetric scores

Group	N	Arithmetic Mean	SD	Median	25th centile	75th centile	Min	Max
Control	35	18	30	10	5	19	2	160
Intervention	35	19	24	12	5	25	2	105

During the study 14 of the intervention group's references had additional hyperlinked PubMed IDs added, most probably by Wikipedia's automatic updating service bots. Also, six of the 35 intervention group *tables* were removed after 2 months (3 pages), 5, 8 and 11 months (1 page each) but the information in the tables remained within the text as did the hyperlinks. As mentioned before, seven of the control pages did already have a reference to the relevant Cochrane review. This was not removed but no table was added.

One review in the control arm had very high page views (25794, 68x the median for whole sample) but not full text accesses (Treatments for delusional disorder(37)) and one review in the intervention arm had very high full text accesses (7407, 18x the median for whole sample; First rank symptoms for schizophrenia(38)).

For peer review only

Although the point estimates for the ratio of geometric means favoured the intervention group for both co-primary outcomes, the confidence intervals were wide and there was no statistical evidence of an effect (Table 3). Results were similar for secondary outcomes, with the exception of Altmetric score which indicated some evidence of an intervention effect, with 95% confidence interval ranging from 5%-78% increase in geometric mean.

For peer review only

Table 3. Results

Group	N	Arithmetic Mean	SD	Geometric Mean	Adjusted ratio of geometric means	95% confidence interval		p-value
**Co-primary outcomes**								
Full text access								
Control	35	654	721	331	-	-	-	
Intervention	35	994	1448	437	1.30	0.71	2.38	0.39
Page views								
Control	35	1427	4379	318	-	-	-	
Intervention	35	618	656	366	1.14	0.60	2.13	0.69
**Secondary outcomes**								
Altmetric score								
Control	35	19	29	11	-	-	-	
Intervention	35	25	32	15	1.36	1.05	1.78	0.02
Abstract views								
Control	35	364	368	228	-	-	-	
Intervention	35	441	464	271	1.17	0.76	1.81	0.47
Unique page views								
Control	35	1307	4032	290	-	-	-	
Intervention	35	561	596	331	1.13	0.60	2.12	0.70
**Group**	**N**	**Arithmetic Mean**	**SD**		**Adjusted difference in means**	**95% confidence interval**		**p-value**
Time on page (seconds)								
Control	35	165	69	-	-	-	-	
Intervention	35	183	76	-	18.51	-16.06	53.08	0.29

Discussion

This is the first randomised trial of Wikipedia content. Our design balanced needs of end-users, Wikipedia administrators and editors and methodologists. The intervention was the insertion of an evidence table and references (with hyperlinks) to the source systematic reviews. This intervention resulted in no clear, statistically significant, difference in access to the full review and page views after one year. All outcome measures consistently favoured a finding indicating increasing activity on the reviews in the ‘intervention’ group although only the Altmetric score – a measure of relevant social media activity – reached conventional levels of statistical significance.

We know of no other randomised trials of Wikipedia content. We previously conducted an RCT of Cochrane Schizophrenia review engagement after sending short messages containing review titles or pertinent questions/results relevant to the review via the social media platforms Twitter and Weibo(39). In that study, the primary outcome of increasing views of the review summary page was met, as were several secondary outcomes measuring review engagement (although we did not have data on full text access or Almetric scores). Importantly, the Twitter study measured further review engagement after people had received a very short fragment (140 characters) of review information, whereas in the current trial we measured engagement after providing people with much more evidence (a concise summary-of-findings table). The embedded summary-of-findings table may have satiated more readers' appetites for evidence. In the Twitter trial, the 'target' page – activity on which triggered recording of outcome - was one click away. In this Wikipedia trial, the reader had to undertake a minimum of two clicks. Although this difference in activity does not sound great, it does indicate a considerable commitment of the reader to pursue more information. For an outcome to be recorded the Wikipedia user had usually to scroll down to find the table, click to expand the drop-down format of the table, seek the reference to that table, and finally click out on one of the hyperlinks. This complex set of actions would, we suggest, indicate high levels of motivation to seek further information and it would seem likely that many users of the Wikipedia pages would have not gone further than the initial page.

There were two outlying, highly-accessed reviews (one in each group). *Post hoc* consideration of causes for this are difficult to avoid. Delusional disorder is a relatively rare but, when encountered, highly worrying condition for clinicians and carers but the review confirms, probably, what most doctors suspect – that there are almost no data from trials to assist decision making(37). This is clearly stated in the abstract as found on the summary page. Further reading may not have been seen as likely to be very rewarding. On the other hand, the diagnostic test review of first rank symptoms was a detailed investigation of the value of one of the foundations of modern psychiatry and, in this case, details in the full text continue to be relevant to daily care(38).

Six tables were deleted at different points across the year out of the 35 inserted into Wikipedia pages. Deletion was undertaken after debate with Wikipedia user and then the

For peer review only

Wikipedia Administrator and is part of the evolution of Wikipedia pages. Administrators have to ensure that this is undertaken in a balanced way taking into account the needs of the readership. Although the tables were deleted, the tables' evidence continued to be reported, as were the hyperlinks. To some readers the tabular format was unacceptable as they felt that tables made the pages "too academic" in appearance. We felt, however, the table was attractive and informative and might encourage interest, seeking of the hyperlink and using it (our primary outcome). Although, after these edits, the hyperlink remained, we think deletion of the table would probably help approximate the results of experimental and control groups.

The addition of the PubMed IDs broadens the options for gaining additional information for the users of the Wikipedia page – again serving to narrow any difference between intervention and control in this trial. Finally, at the very start of the trial, seven of the *control* pages already had some reference to the Cochrane review. We did not feel it right to delete this work for the sake of the trial but the presence of this reference may also have served to narrow the gap between intervention and control groups.

Evaluating techniques of dissemination of knowledge is entirely possible and, with calls for efficient use of ever-more platforms, urgent. Much effort and good will may well be squandered on attractive but ineffective ideas. This first trial of Wikipedia provides enough evidence to suggest more evaluative studies are needed of this particular platform. All outcomes did favour reviews allocated to the Wikipedia page - there was a consistent 13-36% increase in activity across all findings. We think this supports the hypothesis that seeding Wikipedia with evidence could be a potent way of encouraging readers to seek more in-depth information on the effects of care. The hit-rate on the 70 very highly specialised Wikipedia pages was approximately 0.5m/month. If even half were the activity of robotic automated systems(40) that still leaves considerable activity from interested people. How best to seed good evidence into Wikipedia, how best to communicate with this readership, how to use images and infographics, and how to work with Wikipedia to best advantage of all, all are possible to evaluate in future research.

Conclusions

The care Wikipedia invests in the contents of health pages is considerable and the 'live' 'crowd-sourced' and adjudicated peer reviewing of pages is impressive. The outcomes we

For peer review only

were able to record are likely to be only the tip of the ‘activity iceberg’ and placing evidence within Wikipedia seems likely to raise considerably the profile of – in this case – the effects of care.

For peer review only

Trial organisation

The trial was sponsored by the Nottinghamshire Healthcare NHS Foundation Trust. We had no clear reasons to establish a Data Monitoring Committee or a Steering Committee.

Trial registration

IRCT2017070330407N2

Acknowledgements

The research team is grateful to Julie Wood and the Communications and External Affairs team of the Cochrane Collaboration who gave permission for use of the access code for the summaries pages via Google Analytics.

Thanks also to Joanne Thomas and Max Goldman (Sense about Science - senseaboutscience.org) who helped with the language to be used within the tables.

We also wish to thank James Heilman (Wikipedian, The Wikipedia Open Textbook of Medicine) for his ongoing support in seeding Wikipedia with good evidence.

Data sharing

All data from this study are available as Supplementary file 01.

Protocol

https://doi.org/10.22541/au.149926363.33383675

Copyright

The Corresponding Author has the right to grant on behalf of all authors and does grant on behalf of all authors, a worldwide licence to the Publishers and its licensees in perpetuity, in all forms, formats and media (whether known now or created in the future), to i) publish, reproduce, distribute, display and store the Contribution, ii) translate the Contribution into other languages, create adaptations, reprints, include within collections and create summaries, extracts and/or, abstracts of the Contribution, iii) create any other derivative work(s) based on the Contribution, iv) to exploit all subsidiary rights in the Contribution, v) the inclusion of electronic links from the Contribution to third party material where-ever it may be located; and, vi) licence any third party to do any or all of the above.

For peer review only

Competing interest statement

All authors have completed the Unified Competing Interest form (available on request from the corresponding author) and declare: no support from any organisation for the submitted work [or describe if any]; no financial relationships with any organisations that might have an interest in the submitted work in the previous three years [or describe if any], no other relationships or activities that could appear to have influenced the submitted work [or describe if any].

Transparency declaration

The lead author (the manuscript's guarantor) affirms that the manuscript is an honest, accurate, and transparent account of the study being reported; that no important aspects of the study have been omitted; and that any discrepancies from the study as planned (and, if relevant, registered) have been explained.

Details of ethical approval (or a statement that it was not required).

This study employed inanimate Cochrane systematic reviews as participants and collected routine data from electronic systems for outcomes. We enquired of the local Ethics Committee and were advised that ethical approval is not required.

Details of funding

This study was funded by the UK Economic and Social Research Council (ESRC) Impact *Accelerator Fund* (£7.5K) with intramural support from the University of Nottingham.

Statement of independence of researchers from funders

The researchers have no relationship with the ESRC.

Patient and public involvement statement

We did not have patient involvement. However, we did have the involvement of the public. The protocol was drawn up by a group of Wikipedia users – medical and informatics students. In March 2017 we organised a one day meeting to support consultation meeting with students for this trial. This was funded by ESRC (£2.5K of the total described above specifically for this meeting).

The meeting, led by methodologists, also had attendance of representatives of the publisher of the Cochrane Library (John Wiley Ltd.) and of Wikipedia. However, the primary purpose of the day was to get consultation on how the trial should be undertaken from the perspective of one end-user group of Wikipedia – the students. They have continued to be involved in the drafting and writing of the protocol, the conduct of the trial and this final draft report.

Trial registration details (registry and number)

This appears at the end of the abstract (including hyperlink). Recognising that registration is important to help consideration by the major journals, we sought this registration early on – at protocol stage. We were informed that we could not register, as we were not randomising human beings. Because Cochrane Schizophrenia's Information Specialist is from Iran, he knew that local registries do not apply this rule and that key local registries also are uploaded into the international systems – and this includes the registry from Iran – hence why this study is registered in Iran.

Patient consent

Not applicable

Contributorship statement

Authors

Every author contributed – in substantial measure - to the planning, conduct and reporting of the trial. In particular: Clive E Adams, envisioned and led the project and gained [modest] funding for it; Alan A Montgomery helped led the project and undertook the analyses; Tony Aburrow greatly assisted data acquisition from John Wiley; Johannes Friedel and Lena Schmidt undertook software design (SEED) and both also particularly helped with data acquisition from Google Analytics; and Doug Taylor provided continual help with the Wikipedia perspective.

Contributions

Julie Wood and the Communications and External Affairs team of the Cochrane Collaboration gave permission for use of the access code for the summaries pages via Google Analytics.

Joanne Thomas and Max Goldman (Sense about Science - senseaboutscience.org) helped with the language to be used within the tables.

James Heilman (Wikipedian, The Wikipedia Open Textbook of Medicine) supported seeding Wikipedia with good evidence.

For peer review only

References

1. Wikipedia. Wikipedia:About [Internet]. Wikipedia:About. 2016 [cited 2016 Aug 4]. Available from: https://en.wikipedia.org/wiki/Wikipedia:About

2. Wikipedia:Size of Wikipedia. In: Wikipedia [Internet]. 2018 [cited 2018 Dec 3]. Available from: https://en.wikipedia.org/w/index.php?title=Wikipedia:Size_of_Wikipedia&oldid=871450371

3. Monica, erson, Hitlin P, Atkinson M. Wikipedia at 15: Millions of readers in scores of languages [Internet]. Pew Research Center. [cited 2018 Dec 3]. Available from: http://www.pewresearch.org/fact-tank/2016/01/14/wikipedia-at-15/

4. Wikimedia Statistics - All wikis [Internet]. [cited 2018 Dec 3]. Available from: https://stats.wikimedia.org/v2/#/all-projects

5. Alexa Top 500 Global Sites [Internet]. [cited 2017 Apr 10]. Available from: http://www.alexa.com/topsites

6. Heilman J, Bonert M, Beards G, Thomas B, Vondracek A, Grover S, et al. Wikipedia: A Key Tool for Global Public Health Promotion. J Med Internet Res [Internet]. 2011 Mar [cited 2016 Aug 4];13(1). Available from: http://www.jmir.org/2011/1/e14/?trendmd-shared=1

7. Wikipedia. Health information on Wikipedia [Internet]. Health information on Wikipedia. 2016 [cited 2016 Aug 4]. Available from: https://en.wikipedia.org/wiki/Health_information_on_Wikipedia

8. Makovsky. Online Health Research Eclipsing Patient-Doctor Conversations [Internet]. 2013 [cited 2016 Apr 8]. Available from: http://www.makovsky.com/news/online-health-research-eclipsing-patient-doctor-conversations/

9. Laurent M, Vickers T. Seeking health information online: does Wikipedia matter? J Am Med Inform Assoc. 2009 Jul;16(4):471–9.

10. Heilman J, West A. Wikipedia and Medicine: Quantifying Readership, Editors, and the Significance of Natural Language. J Med Internet Res [Internet]. 2015 Mar [cited 2016 Aug 4];17(3). Available from: https://www.ncbi.nlm.nih.gov/pmc/articles/PMC4376174/

11. Hughes B, Joshi I, Lemonde H, Wareham J. Junior physician's use of Web 2.0 for information seeking and medical education: a qualitative study. Int J Med Inf. 2009 Oct;78(10):645–55.

12. Hughes B, Wareham J, Joshi I. Doctors' online information needs, cognitive search strategies, and judgments of information quality and cognitive authority: How predictive judgments introduce bias into cognitive search models. J Assoc Inf Sci Technol. 2010 Mar;61(3):433–52.

13. Rajagopalan M, Khanna V, Leiter Y, Stott M, Showalter T, Dicker A, et al. Patient-Oriented Cancer Information on the Internet: A Comparison of Wikipedia and a Professionally Maintained Database. J Oncol Pract. 2011 Sep;7(5):319–23.

14. Hasty RT, Garbalosa RC, Barbato VA, Valdes PJ, Powers DW, Hernandez E, et al. Wikipedia vs peer-reviewed medical literature for information about the 10 most costly medical conditions. J Am Osteopath Assoc. 2014 May;114(5):368–73.

15. Leithner A, Maurer-Ertl W, Glehr M, Friesenbichler J, Leithner K, Windhager R. Wikipedia and osteosarcoma: a trustworthy patients' information? J Am Med Inform Assoc JAMIA. 2010 Aug;17(4):373–4.

16. Azer SA, AlSwaidan NM, Alshwairikh LA, AlShammari JM. Accuracy and readability of cardiovascular entries on Wikipedia: are they reliable learning resources for medical students? BMJ Open. 2015;5(10):e008187.

17. Phillips J, Lam C, Palmisano L. Analysis of the accuracy and readability of herbal supplement information on Wikipedia. J Am Pharm Assoc JAPhA. 2014 Aug;54(4):406–14.

18. Azer SA. Evaluation of gastroenterology and hepatology articles on Wikipedia: are they suitable as learning resources for medical students? Eur J Gastroenterol Hepatol. 2014 Feb;26(2):155–63.

19. Reavley NJ, Mackinnon AJ, Morgan AJ, Alvarez-Jimenez M, Hetrick SE, Killackey E, et al. Quality of information sources about mental disorders: a comparison of Wikipedia with centrally controlled web and printed sources. Psychol Med. 2012 Aug;42(8):1753–62.

20. Thomas GR, Eng L, de Wolff JF, Grover SC. An evaluation of Wikipedia as a resource for patient education in nephrology. Semin Dial. 2013 Apr;26(2):159–63.

For peer review only

21. Aldairy T, Laverick S, McIntyre GT. Orthognathic surgery: is patient information on the Internet valid? Eur J Orthod. 2012 Aug;34(4):466–9.

22. Reilly T, Jackson W, Berger V, Candelario D. Accuracy and completeness of drug information in Wikipedia medication monographs. J Am Pharm Assoc JAPhA. 2017 Apr;57(2):193-196.e1.

23. Kräenbring J, Monzon Penza T, Gutmann J, Muehlich S, Zolk O, Wojnowski L, et al. Accuracy and completeness of drug information in Wikipedia: a comparison with standard textbooks of pharmacology. PloS One. 2014;9(9):e106930.

24. Clauson KA, Polen HH, Boulos MNK, Dzenowagis JH. Scope, completeness, and accuracy of drug information in Wikipedia. Ann Pharmacother. 2008 Dec;42(12):1814–21.

25. Azer SA. Is Wikipedia a reliable learning resource for medical students? Evaluating respiratory topics. Adv Physiol Educ. 2015 Mar;39(1):5–14.

26. Higgins J, Green S, editors. Cochrane Handbook for Systematic Reviews of Interventions. In: 5.1.0. The Cochrane Collaboration; 2011 [cited 2016 Aug 4]. p. 1.2.2 What is a systematic review? Available from: http://handbook.cochrane.org/chapter_1/1_2_2_what_is_a_systematic_review.htm

27. The Cochrane Library [Internet]. 2014 [cited 2016 Aug 4]. Available from: http://www.cochranelibrary.com/help/access-options-for-cochrane-library.html

28. McIlwain C, Santesso N, Simi S, Napoli M, Lasserson T, Welsh E, et al. Standards for the reporting of Plain Language Summaries in new Cochrane Intervention Reviews (PLEACS) [Internet]. 2013 [cited 2016 Aug 4]. Available from: http://methods.cochrane.org/sites/default/files/uploads/PLEACS_0.pdf

29. Wikipedia. Wikipedia:Cochrane Collaboration/Cochrane UK/About [Internet]. Wikipedia:Cochrane Collaboration/Cochrane UK/About. 2016 [cited 2016 Aug 9]. Available from: https://en.wikipedia.org/wiki/Wikipedia:Cochrane_Collaboration/Cochrane_UK/About

30. White D, Adams CE. What proportion of Cochrane Reviews are Wiki-compatible? | Cochrane Community [Internet]. [cited 2017 Apr 10]. Available from: /news/what-proportion-cochrane-reviews-are-wiki-compatible

For peer review only

31. Adams CE, Montgomery AA, Aburrow A, Bloomfield S, Briley P, Carew E, et al. The effects of Wikipedia referencing: a protocol for a randomised trial. Authorea Prepr [Internet]. 2017 Jul 5 [cited 2017 Oct 12]; Available from: https://www.authorea.com/users/163403/articles/185228-the-effects-of-wikipedia-referencing-a-protocol-for-a-randomised-trial

32. GRADEpro | GDT [Internet]. [cited 2017 Apr 10]. Available from: https://gradepro.org/

33. Google Team. Google Analytics [Internet]. 2014 [cited 2014 Jul 2]. Available from: http://www.google.com/analytics/

34. Schmidt L, Friedel J, Adams CE. SEED: a tool for disseminating systematic review data into Wikipedia. Syst Rev. 2017 Oct 17;6(1):206.

35. Quetiapine. In: Wikipedia [Internet]. 2019 [cited 2019 Jun 11]. Available from: https://en.wikipedia.org/w/index.php?title=Quetiapine&oldid=894522053

36. The Importance of Altmetrics: A Primer | Wiley [Internet]. [cited 2018 Dec 12]. Available from: https://hub.wiley.com/community/exchanges/discover/blog/2016/06/01/the-importance-of-altmetrics-a-primer

37. Skelton M, Khokhar WA, Thacker SP. Treatments for delusional disorder. Cochrane Database Syst Rev. 2015 May 22;(5):CD009785.

38. Soares-Weiser K, Maayan N, Bergman H, Davenport C, Kirkham AJ, Grabowski S, et al. First rank symptoms for schizophrenia. Cochrane Database Syst Rev. 2015;1:CD010653.

39. Adams CE, Jayaram M, Bodart AYM, Sampson S, Zhao S, Montgomery AA. Tweeting links to Cochrane Schizophrenia Group reviews: a randomised controlled trial. BMJ Open. 2016;6(3):e010509.

40. Malicious bot traffic climbs 9.5 percent in 2017, says report [Internet]. SC Media. 2018 [cited 2019 May 21]. Available from: https://www.scmagazine.com/home/research/malicious-bot-traffic-climbs-9-5-percent-in-2017-says-report/

For peer review only

Figure Legend

Figure 1: Sample of embedded table

Figure 2. CONSORT flow diagram

For peer review only

Paliperidone palmitate long-acting injection compared to risperidone for schizophrenia[2]			
When flexibly dosed every four weeks, paliperidone palmitate appears comparable in efficacy and tolerability to risperidone. In short-term studies, paliperidone palmitate – the longer-acting injection – has a similar adverse effect profile to related compounds such as risperidone by mouth. No difference was found in the high rate of reported adverse sexual outcomes and paliperidone palmitate is associated with an increase in serum prolactin.[2]			
[hide] **Outcome**	**Findings in words**	**Findings in numbers**	**Quality of evidence**
**Global state: No clinically important change**			
No 30% improvement on PANSS score. Follow-up: 13-53 weeks	There is no clear difference between people given paliperidone palmitate and those receiving risperidone for this outcome. These findings are based on data of low quality.	RR 1.03 (0.93 to 1.14)	Low
**Relapse**			
Recurrence of psychotic symptoms. Follow up: 13-53 weeks	There is no clear difference between people given paliperidone palmitate and those receiving risperidone for the outcome of 'relapse'. Data supporting this finding are based on moderate quality evidence.	RR 1.23 (0.98 to 1.53)	Moderate
**Leaving the study early**			
- For any reason. Follow up: 13-53 weeks	Paliperidone palmitate causes little or no increase to the chance of leaving the study.	RR 1.12 (1 to 1.25)	High

Figure 1: Example of embedded table

221x165mm (96 x 96 DPI)

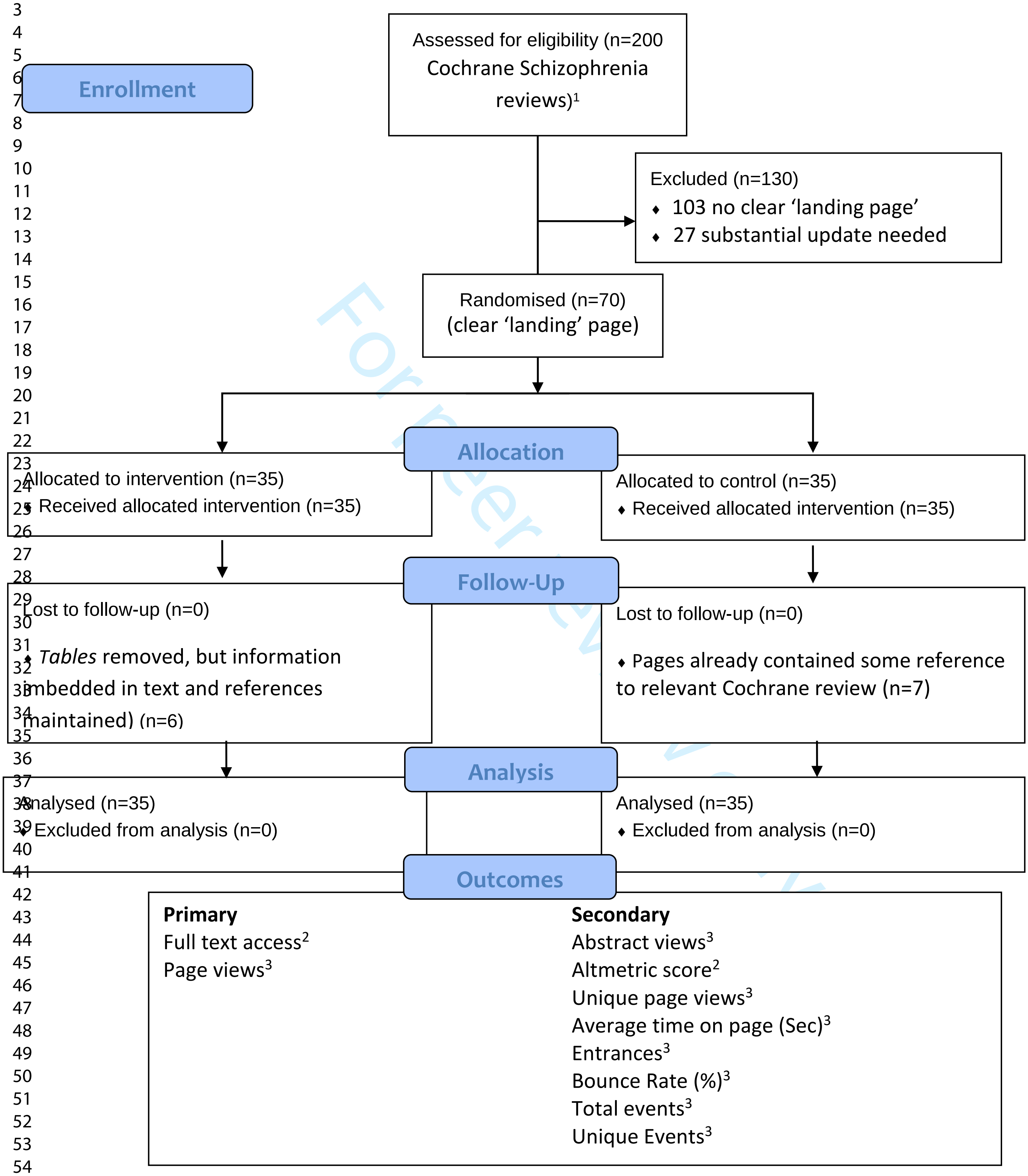

1. 2016 **Data source** 2. John Wiley Ltd 3. Google Analytics

ID	arm	label	Title	Review-URL	DOI	DOI-2	Pageviews	que pagevi
1	0	CONTROL	Acetylchol	https://w	10.1002/1		127	120
2	0	CONTROL	Acupunct	https://w	10.1002/1		753	670
4	0	CONTROL	Amisulpri	https://w	10.1002/1		351	307
5	1	WIKIPEDI	Aripiprazo	https://w	10.1002/1		1132	1009
6	1	WIKIPEDI	Art	https://w	10.1002/1		902	801
7	1	WIKIPEDI	Asenapine	https://w	10.1002/1		116	106
9	0	CONTROL	Benzodiaz	https://w	10.1002/1		822	722
11	0	CONTROL	Chlorpro	https://w	10.1002/1		165	152
12	1	WIKIPEDI	Clotiapine	https://w	10.1002/1		194	175
13	0	CONTROL	Clozapine	https://w	10.1002/1		831	753
14	1	WIKIPEDI	Cognitive	https://w	10.1002/1		822	754
15	0	CONTROL	Cognitive	https://w	10.1002/1		230	199
16	0	CONTROL	Communit	https://w	10.1002/1		49	47
17	1	WIKIPEDI	Complianc	https://w	10.1002/1		562	509
18	0	CONTROL	Dance	https://w	10.1002/1		683	602
19	0	CONTROL	Treatment	https://w	10.1002/1		25794	23739
20	1	WIKIPEDI	First rank	https://w	10.1002/1		952	892
22	0	CONTROL	Droperido	https://w	10.1002/1		221	199
23	1	WIKIPEDI	Early	https://w	10.1002/1		1046	959
24	1	WIKIPEDI	Education	https://w	10.1002/1		639	588
25	0	CONTROL	Estrogen	https://w	10.1002/1		123	110
26	0	CONTROL	Electrocon	https://w	10.1002/1		1302	1164
27	0	CONTROL	Crisis	https://w	10.1002/1		719	640
29	0	CONTROL	Intercesso	https://w	10.1002/1		607	548
31	0	CONTROL	Fluphenaz	https://w	10.1002/1		49	45
32	1	WIKIPEDI	Depot	https://w	10.1002/1		53	49
33	1	WIKIPEDI	Glutamate	https://w	10.1002/1		528	480
34	1	WIKIPEDI	Haloperid	https://w	10.1002/1		422	393
35	1	WIKIPEDI	Horticultu	https://w	10.1002/1		318	289
36	1	WIKIPEDI	Pharmacol	https://w	10.1002/1		419	381
38	0	CONTROL	Compulso	https://w	10.1002/1	10.1002/1	1053	952
40	1	WIKIPEDI	Levomepr	https://w	10.1002/1		141	128
41	1	WIKIPEDI	Life skills	https://w	10.1002/1		881	795
42	1	WIKIPEDI	Cannabis	https://w	10.1002/1		1089	1004
43	0	CONTROL	Loxapine	https://w	10.1002/1		244	226
44	1	WIKIPEDI	Antioxida	https://w	10.1002/1		492	449
45	0	CONTROL	Antiglucoc	https://w	10.1002/1		136	131
46	0	CONTROL	Molindon	https://w	10.1002/1		33	33
47	0	CONTROL	Lithium	https://w	10.1002/1		4592	4227
49	1	WIKIPEDI	Music	https://w	10.1002/1	10.1002/1	2508	2264
52	1	WIKIPEDI	Paliperido	https://w	10.1002/1		274	252
53	1	WIKIPEDI	Atypical	https://w	10.1002/1		260	241
54	1	WIKIPEDI	Penflurido	https://w	10.1002/1		152	112
55	1	WIKIPEDI	Perazine	https://w	10.1002/1		106	95
56	1	WIKIPEDI	Pericyazin	https://w	10.1002/1		88	82
57	1	WIKIPEDI	Perphenaz	https://w	10.1002/1		326	299
59	1	WIKIPEDI	Pimozide	https://w	10.1002/1		59	54
60	1	WIKIPEDI	Depot	https://w	10.1002/1		41	39
63	0	CONTROL	Advance	https://w	10.1002/1		101	97

64	1	WIKIPEDI	Supported	https://w	10.1002/1		444	397
65	0	CONTROL	Individual	https://w	10.1002/1		394	355
66	0	CONTROL	Psychoed	https://w	10.1002/1		727	660
67	0	CONTROL	Psychosoc	https://w	10.1002/1		647	602
69	0	CONTROL	Pyridoxal	https://w	10.1002/1		80	72
70	1	WIKIPEDI	Quetiapin	https://w	10.1002/1		340	306
71	1	WIKIPEDI	Risperidon	https://w	10.1002/1		371	324
73	1	WIKIPEDI	Intensive	https://w	10.1002/1		2738	2489
76	0	CONTROL	Sertindole	https://w	10.1002/1		105	95
77	0	CONTROL	Managem	https://w	10.1002/1		4052	3810
79	0	CONTROL	Social	https://w	10.1002/1		613	559
81	1	WIKIPEDI	Supportiv	https://w	10.1002/1		1012	915
82	0	CONTROL	Vitamin E	https://w	10.1002/1	10.1002/1	688	620
83	0	CONTROL	Thioridazi	https://w	10.1002/1		30	25
84	1	WIKIPEDI	Token	https://w	10.1002/1		128	113
85	0	CONTROL	Transcrani	https://w	10.1002/1		3457	3116
86	1	WIKIPEDI	Trifluoper	https://w	10.1002/1		97	85
87	1	WIKIPEDI	Valproate	https://w	10.1002/1		1979	1808
89	0	CONTROL	Ziprasidon	https://w	10.1002/1		31	25
90	0	CONTROL	Zotepine	https://w	10.1002/1		13	13
91	0	CONTROL	Zuclopent	https://w	10.1002/1		119	107

For peer review only

me on page	Entrances	unce Rate (	Total events	nique Even	ll text acce	bstract view	tmetric sco	ews mentio
175	60	73,33	105	105	164	190	8	0
93	191	68,59	0	0	813	238	32	0
144	221	72,20	263	263	83	105	6	0
179	833	70,97	0	0	1181	761	31	0
176	573	65,98	158	158	749	1001	25	0
150	75	64	0	0	229	120	30	0
153	545	79,30	631	526	252	232	9	0
252	123	74,80	53	53	469	216	26	0
91	126	81,75	0	0	64	137	7	0
141	444	67,85	421	421	512	422	15	0
196	517	67,94	105	105	1667	1261	86	1
55	37	59,46	0	0	245	129	4	0
188	21	63,64	0	0	734	270	4	0
189	417	76,56	368	263	398	273	16	1
208	394	65,99	263	158	1260	577	24	1
227	21080	84,66	2629	2366	2044	1792	39	1
384	824	82,42	0	0	7407	584	28	0
143	125	68,22	263	263	533	277	10	0
194	644	56,57	631	631	1699	927	75	1
122	428	79,02	210	158	205	141	14	0
78	67	77,61	0	0	81	73	3	0
155	735	68,07	315	315	1262	919	101	12
157	363	75,62	158	158	2211	705	13	0
212	437	79.5	105	105	364	729	155	8
57	26	76,92	0	0	138	59	14	0
140	37	73,17	105	105	54	51	5	0
104	344	82,56	53	53	143	99	13	0
225	265	80,15	0	0	516	212	14	0
101	112	73,45	0	0	1415	509	7	0
115	147	82,99	0	0	254	385	6	0
279	784	68,05	473	473	1436	766	16	0
157	109	78,90	0	0	49	72	6	0
200	609	74,80	158	158	1301	613	11	0
126	398	69,31	210	210	3794	978	93	6
150	181	75,41	0	0	99	142	7	0
151	231	70,26	158	105	864	392	17	0
177	84	79,31	0	0	301	143	6	0
82	18	94,44	0	0	13	54	4	0
256	4064	86,59	263	219	835	254	21	0
211	1777	77,19	1209	1104	3241	2297	152	1
256	205	70,81	158	158	252	322	19	0
175	162	73,62	0	0	727	393	15	0
121	99	65,66	158	158	72	191	9	0
173	81	80,25	0	0	159	54	5	0
161	66	84,85	0	0	130	73	6	0
233	281	75,80	210	158	311	116	6	0
116	42	66,67	0	0	376	120	17	0
448	27	66,67	0	0	21	46	8	0
259	67	65,67	0	0	425	228	6	0

For peer review only

261	289	67,93	158	158	1653	593	32	1
107	207	68,05	0	0	454	256	16	0
217	533	73,84	53	53	1228	885	12	0
181	305	67,83	315	315	3155	951	17	0
115	20	59,09	0	0	280	112	5	0
142	182	76,09	53	0	406	180	11	0
122	182	65,03	683	368	223	233	7	0
252	2151	80,06	736	683	2569	1062	64	0
173	82	74,39	0	0	34	36	4	0
280	3702	86,20	841	631	329	376	8	0
308	424	74,36	105	105	1455	484	9	0
205	726	79,26	158	158	1583	501	10	0
104	548	77,41	0	0	407	378	11	0
56	19	73,68	0	0	50	108	5	0
119	98	84,68	0	0	262	261	5	0
198	2835	82,06	53	53	927	463	27	0
166	73	76,71	0	0	171	62	20	0
256	1647	80,89	473	473	649	402	9	0
170	15	53,33	105	105	54	83	10	0
66	6	66,67	0	0	25	21	2	0
145	60	73,33	0	0	207	63	10	0

For peer review only

log mentio	licy mentio	itter menti	tent menti	review mer	eibo menti	ebook ment	pedia ment	gle+ menti
0	0	16	0	0	0	2	2	0
1	0	35	0	0	3	7	2	3
0	0	14	0	0	0	0	3	0
2	1	52	0	0	2	0	3	0
0	0	60	0	0	2	1	4	1
0	0	48	0	0	0	3	1	1
0	0	20	0	0	3	2	0	0
3	0	37	0	1	3	1	0	0
0	0	28	0	0	2	0	1	0
1	0	24	0	0	2	1	0	1
7	0	103	0	0	1	1	2	1
0	0	33	0	0	3	0	0	0
0	0	17	0	0	0	0	0	0
0	0	12	0	0	3	0	1	0
1	0	14	0	0	3	0	0	0
2	0	39	0	0	0	7	0	0
1	0	38	0	0	0	1	1	0
0	0	19	0	0	0	2	0	0
6	1	37	0	0	0	1	5	0
1	0	12	0	0	0	0	1	0
0	0	11	0	0	0	0	0	0
0	0	24	0	0	0	0	1	0
1	0	17	0	0	0	3	0	0
9	0	59	0	0	0	8	2	1
1	0	14	0	0	0	0	1	0
0	0	15	0	0	3	0	1	0
1	0	13	0	0	0	0	1	0
1	0	9	0	0	0	1	2	0
0	0	18	0	0	0	2	0	0
0	0	12	0	0	0	0	1	0
0	0	34	0	0	0	4	3	1
0	0	11	0	0	0	0	1	0
0	0	23	0	0	0	1	1	1
2	0	41	0	0	0	1	2	2
0	0	32	0	0	2	0	2	0
0	0	30	0	0	0	3	1	1
0	0	10	0	0	0	1	0	0
0	0	10	0	0	0	0	1	0
1	0	37	0	0	0	0	0	0
0	0	216	0	0	0	12	1	0
1	0	38	0	0	2	1	1	0
1	0	16	0	0	0	0	1	0
0	0	23	0	0	3	0	1	0
0	0	10	0	0	0	1	2	0
0	0	12	0	0	0	1	2	0
0	0	13	0	0	0	2	2	0
1	0	29	0	0	3	1	2	0
0	0	27	0	0	3	0	1	0
0	1	14	0	0	0	0	0	0

For peer review only

0	1	33	0	0	0	1	1	0
0	0	25	0	0	2	0	1	0
0	0	33	0	0	0	0	0	0
1	0	25	0	0	0	1	0	0
0	0	11	0	0	0	1	1	0
0	0	18	0	0	0	2	1	0
0	0	22	0	0	0	1	1	0
0	0	99	0	0	0	0	2	0
0	0	17	0	0	3	0	0	0
0	0	28	0	0	3	0	0	0
0	0	16	0	0	0	1	0	1
0	0	18	0	0	1	0	1	0
0	0	14	0	0	0	1	1	0
0	0	5	0	0	3	0	2	0
0	0	13	0	0	0	0	1	0
1	0	37	0	0	0	1	4	0
1	0	26	0	0	2	1	1	0
0	0	15	0	0	0	2	1	0
1	0	14	0	0	0	0	0	0
0	0	9	0	0	0	1	0	0
0	0	18	0	0	0	1	0	0

For peer review only

kedIn menti	ddit menti	erest ment	000 mentio	&A mentio	deo mentio	labi mentio	of Mendele	Dimension
0	0	0	0	0	0	0	100	23
0	0	0	0	0	0	0	167	9
0	0	0	0	0	0	0	89	25
0	0	0	0	0	0	0	200	45
0	0	0	0	0	0	0	8	53
0	0	0	0	0	0	0	55	5
0	0	0	0	0	0	0	1	33
0	0	0	0	0	1	0	185	36
0	0	0	0	0	0	0	57	19
0	0	0	0	0	0	0	136	114
0	0	0	0	0	0	0	82	86
0	0	0	0	0	0	0	13	43
0	0	0	0	0	0	0	34	80
0	0	0	0	0	0	0	64	55
0	0	0	0	0	0	0	55	28
0	0	0	0	0	0	0	102	11
0	0	0	0	0	0	0	133	12
0	0	0	0	0	0	0	226	11
0	0	0	0	0	0	0	73	143
0	0	0	0	0	0	0	14	31
0	0	0	0	0	0	0	66	51
0	0	0	0	0	0	0	10	156
0	0	0	0	0	0	0	31	14
0	1	0	0	0	2	0	193	50
0	0	0	0	0	0	0	1	7
0	0	0	0	0	0	0	52	13
0	0	0	0	0	0	0	51	60
0	0	0	0	0	0	0	165	24
0	0	0	0	0	0	0	33	6
0	0	0	0	0	0	0	74	24
0	0	0	0	0	0	0	39	13
0	0	0	0	0	0	0	68	10
0	0	0	0	0	0	0	52	20
0	0	0	0	0	0	0	5	23
0	0	0	0	0	0	0	68	39
0	0	0	0	0	0	0	92	26
0	0	0	0	0	0	0	104	12
0	0	0	0	0	0	0	70	6
0	0	0	0	0	0	0	120	15
0	0	0	0	0	0	0	30	17
0	0	0	0	0	0	0	65	30
0	0	0	0	0	0	0	9	26
0	0	0	0	0	0	0	39	9
0	0	0	0	0	0	0	67	4
0	0	0	0	0	0	0	58	4
0	0	0	0	0	0	0	100	2
0	0	0	0	0	0	0	163	15
0	0	0	0	0	0	0	46	8
0	0	0	0	0	0	0	132	47

For peer review only

0	0	0	0	0	0	0	45	80
0	0	0	0	1	0	0	135	45
0	0	0	0	0	0	0	3	139
0	0	0	0	0	0	0	64	81
0	0	0	0	0	0	0	65	7
0	0	0	0	0	0	0	108	19
0	0	0	0	0	0	0	139	67
0	0	0	0	0	0	0	71	19
0	0	0	0	0	0	0	69	15
0	0	0	1	0	0	0	18	33
0	0	0	0	0	0	0	102	17
0	0	0	0	0	0	0	37	7
0	0	0	0	0	0	0	16	2
0	0	0	0	0	0	0	100	23
0	0	0	0	0	0	0	25	21
0	0	0	0	0	0	0	40	15
0	0	0	0	0	0	0	81	7
0	0	0	0	0	0	0	130	12
0	0	0	0	0	0	0	73	55
0	0	0	0	0	0	0	73	6
0	0	0	0	0	0	0	54	1

For peer review only

ıs citations

For peer review only

CONSORT checklist of items for reporting pragmatic trials

Section	Item	Standard CONSORT description	Extension for pragmatic trials	Line
Title and abstract	1	How participants were allocated to interventions (eg, “random allocation,” “randomised,” or “randomly assigned”)		1 (title)
**Introduction**				1
Background	2	Scientific background and explanation of rationale	Describe the health or health service problem that the intervention is intended to address and other interventions that may commonly be aimed at this problem	2-62
**Methods**				66
Participants	3	Eligibility criteria for participants; settings and locations where the data were collected	Eligibility criteria should be explicitly framed to show the degree to which they include typical participants and/or, where applicable, typical providers (eg, nurses), institutions (eg, hospitals), communities (or localities eg, towns) and settings of care (eg, different healthcare financing systems)	79
Interventions	4	Precise details of the interventions intended for each group and how and when they were actually administered	Describe extra resources added to (or resources removed from) usual settings in order to implement intervention. Indicate if efforts were made to standardise the intervention or if the intervention and its delivery were allowed to vary between participants, practitioners, or study sites	100 (+ Figure 1)

For peer review

Section	Item	Standard CONSORT description	Extension for pragmatic trials	Line
			Describe the comparator in similar detail to the intervention	
Objectives	5	Specific objectives and hypotheses		63
Outcomes	6	Clearly defined primary and secondary outcome measures and, when applicable, any methods used to enhance the quality of measurements (eg, multiple observations, training of assessors)	Explain why the chosen outcomes and, when relevant, the length of follow-up are considered important to those who will use the results of the trial	124
Sample size	7	How sample size was determined; explanation of any interim analyses and stopping rules when applicable	If calculated using the smallest difference considered important by the target decision maker audience (the minimally important difference) then report where this difference was obtained	133
Randomisation—sequence generation	8	Method used to generate the random allocation sequence, including details of any restriction (eg, blocking, stratification)		97
Randomisation—allocation concealment	9	Method used to implement the random allocation sequence (eg, numbered containers or central telephone), clarifying whether the sequence was concealed until interventions were assigned		98-99
Randomisation—implementation	10	Who generated the allocation sequence, who enrolled participants, and who assigned participants to their groups		97
Blinding (masking)	11	Whether participants, those administering the interventions, and those assessing the outcomes were blinded to group assignment	If blinding was not done, or was not possible, explain why	115

For peer review only

Section	Item	Standard CONSORT description	Extension for pragmatic trials	Line
Statistical methods	12	Statistical methods used to compare groups for primary outcomes; methods for additional analyses, such as subgroup analyses and adjusted analyses		140
**Results**				147
Participant flow	13	Flow of participants through each stage (a diagram is strongly recommended)—specifically, for each group, report the numbers of participants randomly assigned, receiving intended treatment, completing the study protocol, and analysed for the primary outcome; describe deviations from planned study protocol, together with reasons	The number of participants or units approached to take part in the trial, the number which were eligible, and reasons for non-participation should be reported	Figure 2
Recruitment	14	Dates defining the periods of recruitment and follow-up		109, 125
Baseline data	15	Baseline demographic and clinical characteristics of each group		Table 2
Numbers analysed	16	Number of participants (denominator) in each group included in each analysis and whether analysis was by "intention-to-treat"; state the results in absolute numbers when feasible (eg, 10/20, not 50%)		Figure 2
Outcomes and estimation	17	For each primary and secondary outcome, a summary of results for each group and the estimated effect size and its precision (eg, 95% CI)		Table 3
Ancillary analyses	18	Address multiplicity by reporting any other analyses performed, including subgroup analyses and adjusted analyses, indicating which are prespecified and which are exploratory		N/A - ?198

For peer review only

Section	Item	Standard CONSORT description	Extension for pragmatic trials	Line
Adverse events	19	All important adverse events or side effects in each intervention group		N/A
**Discussion**				168
Interpretation	20	Interpretation of the results, taking into account study hypotheses, sources of potential bias or imprecision, and the dangers associated with multiplicity of analyses and outcomes		168-235
Generalisability	21	Generalisability (external validity) of the trial findings	Describe key aspects of the setting which determined the trial results. Discuss possible differences in other settings where clinical traditions, health service organisation, staffing, or resources may vary from those of the trial	?? 236
Overall evidence	22	General interpretation of the results in the context of current evidence		236

Cite as: *Zwarenstein M, Treweek S, Gagnier JJ, Altman DG, Tunis S, Haynes B, Oxman AD, Moher D for the CONSORT and Pragmatic Trials in Healthcare (Practihc) group. Improving the reporting of pragmatic trials: an extension of the CONSORT statement. BMJ 2008; 337;a2390.*

For peer review only

BMJ Open

The effects of adding evidence of the effects of treatments into relevant Wikipedia pages on further information-seeking behaviour: a randomised trial.

Journal:	*BMJ Open*
Manuscript ID	bmjopen-2019-033655.R1
Article Type:	Research
Date Submitted by the Author:	28-Nov-2019

Complete List of Authors:	Adams, Clive; University of Nottingham, Division of Psychiatry and Applied Psychology<br>Montgomery, Alan; University of Nottingham, Nottingham Clinical Trials Unit<br>Aburrow, Tony; John Wiley and Sons, Limited<br>Bloomfield, Sophie; East Kent Hospitals University NHS Foundation Trust<br>Briley, Paul; University of Nottingham, Division of Psychiatry and Applied Psychology<br>Carew, Ebun; Nottingham University Hospitals Healthcare NHS Trust<br>Chatterjee-Woolman, Suravi; Sherwood Forest Hospitals NHS Foundation Trust<br>Feddah, Ghalia; Sherwood Forest Hospitals NHS Foundation Trust<br>Friedel, Johannes; University of Aalen<br>Gibbard, Josh; Morecambe Bay Hospitals NHS Trust<br>Haynes, Euan; Gateshead Health NHS Foundation Trust<br>Hussein, Mohsin; University Hospitals of Leicester NHS Trust<br>Jayaram, Mahesh; University of Melbourne, Psychaitry<br>Naylor, Samuel; Sherwood Forest Hospitals NHS Foundation Trust<br>Perry, Luke; Royal Melbourne Hospital<br>Schmidt, Lena; University of Bristol Faculty of Health Sciences<br>Siddique, Umer; Barking Havering and Redbridge Hospitals NHS Trust<br>Tabaksert, Ayla; Northumbria Healthcare NHS Foundation Trust<br>Taylor, Douglas; Wikimedia Foundation Inc<br>Velani, Aarti; Nottingham University Hospitals NHS Trust<br>White, Douglas; Epsom and Saint Helier University Hospitals NHS Trust Epsom Hospital<br>Xia, Jun; Nottingham China Health Institute, The University of Nottingham Ningbo

**Primary Subject Heading**:	Health informatics
Secondary Subject Heading:	Medical education and training, Medical publishing and peer review, Mental health
Keywords:	MEDICAL EDUCATION & TRAINING, Schizophrenia & psychotic disorders < PSYCHIATRY, World Wide Web technology < BIOTECHNOLOGY & BIOINFORMATICS

SCHOLARONE™
Manuscripts

I, the Submitting Author has the right to grant and does grant on behalf of all authors of the Work (as defined in the below author licence), an exclusive licence and/or a non-exclusive licence for contributions from authors who are: i) UK Crown employees; ii) where BMJ has agreed a CC-BY licence shall apply, and/or iii) in accordance with the terms applicable for US Federal Government officers or employees acting as part of their official duties; on a worldwide, perpetual, irrevocable, royalty-free basis to BMJ Publishing Group Ltd ("BMJ") its licensees and where the relevant Journal is co-owned by BMJ to the co-owners of the Journal, to publish the Work in this journal and any other BMJ products and to exploit all rights, as set out in our licence.

The Submitting Author accepts and understands that any supply made under these terms is made by BMJ to the Submitting Author unless you are acting as an employee on behalf of your employer or a postgraduate student of an affiliated institution which is paying any applicable article publishing charge ("APC") for Open Access articles. Where the Submitting Author wishes to make the Work available on an Open Access basis (and intends to pay the relevant APC), the terms of reuse of such Open Access shall be governed by a Creative Commons licence – details of these licences and which Creative Commons licence will apply to this Work are set out in our licence referred to above.

Other than as permitted in any relevant BMJ Author's Self Archiving Policies, I confirm this Work has not been accepted for publication elsewhere, is not being considered for publication elsewhere and does not duplicate material already published. I confirm all authors consent to publication of this Work and authorise the granting of this licence.

For peer review only

Title: The effects of adding evidence of the effects of treatments into relevant Wikipedia pages on further information-seeking behaviour: a randomised trial.

Authors:

Clive E Adams, Alan A Montgomery, Tony Aburrow, Sophie Bloomfield, Paul M Briley, Ebun Carew, Suravi Chatterjee-Woolman, Ghalia Feddah, Johannes Friedel, Josh Gibbard, Euan Haynes, Mohsin Hussein, Mahesh Jayaram, Samuel D Naylor, Luke Perry, Lena Schmidt, Umer Siddique, Ayla S Tabaksert, Douglas Taylor, Aarti Velani, Douglas White, Jun Xia

Contact:

Professor Clive E Adams

Chair Mental Health Services Research, Co-ordinating Editor, Cochrane Schizophrenia

Institute of Mental Health

Jubilee Campus

University of Nottingham Innovation Park

Triumph Road

Nottingham

NG7 2TU

Phone: 0115 823 1294

Fax: 0115 823 1294

Email: clive.adams@nottingham.ac.uk

Co-authors:

Alan A Montgomery, Nottingham Clinical Trials Unit, University of Nottingham, Nottingham, UK,
Tony Aburrow, John Wiley Ltd., Chichester, UK,

Sophie Bloomfield, East Kent Hospitals University Foundation Trust, Ashford, UK

Paul M Briley, Institute of Mental Health, University of Nottingham, Nottingham, UK

Ebun Carew, Nottingham University Hospitals Healthcare NHS Trust, Nottingham, UK

For peer review only

Suravi Chatterjee-Woolman, Sherwood Forest Hospitals NHS Foundation Trust, Sutton in Ashfield, UK

Ghalia Feddah, Sherwood Forest Hospitals NHS Foundation Trust, Sutton in Ashfield, UK

Johannes Friedel, University of Aalen, Aalen, Germany

Josh Gibbard, Morecambe Bay Hospitals NHS Trust, Lancaster, UK

Euan Haynes, Gateshead Health, NHS Foundation Trust, Gateshead, UK

Mohsin Hussein, Leicester Evington Centre, Leicester, UK

Mahesh Jayaram, Department of Psychiatry, University of Melbourne, Australia

Samuel D Naylor, Sherwood Forest Hospitals NHS Foundation Trust, Sutton in Ashfield, UK

Luke Perry, Royal Melbourne Hospital, Parkville, Australia

Lena Schmidt, Hochschule Furtwangen University, Furtwangen, Germany; University of Bristol, Bristol, UK

Umer Siddique, Barking, Havering and Redbridge Hospitals NHS Trust, London, UK

Ayla S Tabaksert, Northumbria NHS Foundation Trust, Newcastle upon Tyne, UK

Douglas Taylor, Wikimedia UK, Loughborough, UK

Aarti Velani, Nottingham University Hospitals Healthcare NHS Trust, Nottingham, UK

Douglas White, Epsom and St Helier University Hospitals NHS Trust, London, UK

Jun Xia, Nottingham China Health Institute, The University of Nottingham Ningbo, Ningbo, China

Key words

Medical education

Health Informatics

Psychiatry

Randomized controlled trial

Word count 3207

For peer review only

Abstract

Objectives

To investigate the effects of adding high-grade quantitative evidence of outcomes of treatments into relevant Wikipedia pages on further information-seeking behaviour by use of routinely collected data.

Setting

Wikipedia, Cochrane summary pages and the Cochrane Library.

Design

Randomised trial.

Participants

Wikipedia pages which were highly relevant to up-to-date Cochrane Schizophrenia systematic reviews that contained a Summary of Findings table.

Interventions

Eligible Wikipedia pages in the intervention group were seeded with tables of best evidence of the effects of care and hyperlinks to the source Cochrane review. Eligible Wikipedia pages in the control group were left unchanged.

Main outcome measures

Routinely collected data on access to the full text and summary web-page (after 12 months).

Results

We randomised 70 Wikipedia pages (100% follow up). Six of the 35 Wikipedia pages in the intervention group had the tabular format deleted during the study but all pages continued to report the same data within the text. There was no evidence of effect on either of the co-primary outcomes: full text access adjusted ratio of geometric means 1.30, 95% CI 0.71 to 2.38; page views 1.14, 95% CI 0.6 to 2.13. Results were similar for all other outcomes, with exception of Altmetric score for which there was some evidence of clear effect (1.36, 95% CI 1.05 to 1.78.

For peer review only

Conclusions

For every person who sought and clicked the reference on the Wikipedia page to seek more information (the primary outcome), many more are likely to have been informed by the page alone. Enriching Wikipedia content is, potentially, a powerful way to improve health literacy. The pursuit of fair balance within Wikipedia health care pages is impressive and its reach unsurpassed. It is possible to test the effects of seeding pages with evidence and this trial should be replicated, expanded and developed. [Abstract 300 words]

Trial registration

[Prospectively registered 20/07/2017; IRCT2017070330407N2]

For peer review only

Strengths and limitations of this study

- First randomised trial of placement of evidence within Wikipedia pages
- Use of routine data to allow 100% follow up
- Open editing of Wikipedia pages – both intervention and control pages - by the Wikipedia community served to minimise difference between groups.
- Outcomes necessitated unusual levels of interest and commitment on the part of the Wikipedia page reader
- Small study in highly specialised area of heath care

Background

Wikipedia is a free-content online encyclopaedia containing articles on a vast range of topics(1). At present there are over 5.7m articles, 46 million pages in the English language(2). Since its creation in 2001, Wikipedia has expanded to attract over 27m registered users(3) with 16 billion pages views per month(4). This made Wikipedia the 5th most popular site on the internet in 2017(5).

Wikipedia is openly-editable. This means that any one of these users can access *and edit* the majority of articles. Wikipedia policy states, however, that all information presented in pages must be "verifiable against a published reliable source"(1). Therefore, all pages aim to contain references for the information they provide. To prevent the risk of pages being devalued with misinformation Wikipedia has various quality control measures. These include; a 'watchlist' to notify editors when a page has been edited, a published list of recent changes that editors can access to review, automated computer scripts, page protection on more controversial pages, edit filters on certain pages and blocking any editors who repeatedly damage the value of the page (6). On top of this, Wikipedia has a team of administrators. They are editors who have been given access to additional tools on their account. These include the ability to block/unblock accounts, edit fully protected pages and delete/undelete pages. There are 1,194 administrators on the English language Wikipedia (as of December 2018)(2).

Wikipedia contains many pages relating to healthcare. In 2014 the English language version was estimated to contain 25,000 articles on health-related topics, while across all languages there are 155,000 articles containing 950,000 references(7). These are often accessed via search engine results with one survey suggesting that around 22% of healthcare-related online searches direct to Wikipedia pages(8,9). In 2013 health pages on Wikipedia received 4.8 billion views, making it one of the most used means for accessing health information globally(10). When use of Wikipedia is studied in medical students and doctors, it is clear that it is becoming an increasingly popular resource(11,12). This is, perhaps, enhanced by Wikipedia being entirely free of charge – including data download charges in low and middle income countries. In this context there is criticism that as Wikipedia is openly editable, the information it contains may be unreliable. Some evidence suggests, however, that there is no difference in accuracy when Wikipedia is compared to other professionally maintained

For peer review only

medical databases(13) although opinions differ by subspecialty, depend on the 'target' readership and vary across time (Table 1).

Table 1: Selection of studies of Wikipedia's value to different readerships by medical subspecialty

**Sub-specialty (reference)**	**Date**	**Assessing for suitability for….**	**Conclusion**
**10 most costly conditions (14)**	2014	General readership	Most Wikipedia articles representing the 10 most costly medical conditions [….] contain many errors when checked against standard peer-reviewed sources. Caution should be used […..]
**Cancer – general (13)**	2011	Patients	Wiki resource had similar accuracy and depth as the professionally edited database
**Cancer – osteoscarcoma (15)**	2010	Patients	[…] the quality of osteosarcoma-related information found in the English Wikipedia is good but inferior to the patient information provided by the NCI.
**Cardiovascular (16)**	2015	Medical students	Wikipedia entries are not aimed at a medical audience and should not be used as a substitute to recommended medical resources. Course designers and students should be aware that Wikipedia entries on cardiovascular diseases lack accuracy, predominantly due to errors of omission.
**Complementary medicine (17)**	2014	General readership	Patients and health professionals should not rely solely on Wikipedia for information on these herbal supplements when treatment decisions are being made.
**Gastro – hepatology (18)**	2014	Medical students	…..not good source of evidence
**Mental health (19)**	2012	General readership	The quality of information on depression and schizophrenia on Wikipedia is generally as good as, or better than, that provided by centrally controlled

			websites, Encyclopaedia Britannica and a psychiatry textbook.
**Nephrology (20)**	2013	Patients	Fairly reliable medical resource
**Orthognathic surgery (21)**	2012	Patients	Maximum […] score[ings in comparison to other online sources] were Wikipedia
**Pharmacology (22)**	2017	Doctors	Wikipedia lacks the accuracy and completeness of standard clinical references and should not be a routine part of clinical decision making.
**Pharmacology (23)**	2014	Medical students	… Wikipedia is an accurate and comprehensive source of drug-related information for undergraduate medical education.
**Pharmacology (24)**	2008	Patients	Wikipedia has a more narrow scope, is less complete, and has more errors of omission than the comparator database. Wikipedia may be a useful point of engagement for consumers, but is not authoritative and should only be a supplemental source of drug information.
**Respiratory medicine (25)**	2015	Medical students	Most articles had knowledge deficiencies, were not accurate, and were not suitable for medical students as learning resources.

The Cochrane Collaboration(26) is a non-for profit NGO producing, and maintaining systematic reviews of health care published within the *Cochrane Library (*by John Wiley Ltd.). The Collaboration is made up of subgroups and Cochrane Schizophrenia produces and updates high quality systematic reviews and meta-analyses relevant to people with schizophrenia and related psychotic conditions(27). In 2004 a group called WikiProject Medicine was started with the aim of creating and managing medical articles on Wikipedia. This group allows discussion and collaboration on these articles to improve the quality of the information presented(6). In 2014 a formalised partnership between Wikipedia and Cochrane was created, aiming to "transform the quality and content of health evidence available online"(28). This involves incorporating Cochrane's evidence into Wikipedia articles and improving the information's accuracy and reliability.

For peer review only

Whilst increasing accessibility of highest grade maintained health care information seems a laudable aim, objective quantification of the effects of this effort has not been undertaken. This paper reports a collaboratively designed pragmatic randomised trial of adding evidence of the effects of care to Wikipedia health pages on the routinely collected indicators of readers' interest.

Aims

To evaluate the effects of enriching Wikipedia content with summary tables from level 1 evidence on the effects of care.

Methods

In preliminary work, we tested stability of target pages in Wikipedia. Adding an evidence-table to four Wikipedia pages (trifluoperazine – a less used antipsychotic e.g 3529 ± 198 prescriptions/month - figures are for 2018, NHS England(29); chlorpromazine – a old widely-used antipsychotic drug 22386 ±803 prescriptions/month; palperidone – a expensive new antipsychotic drug 853 ± 34 prescriptions/month, and one important talking therapy - cognitive behavioural therapy). These four pages all remained stable over a 12 month period(2015). Further work investigated what proportion of the topics of Cochrane Schizophrenia reviews already had a highly specific page in Wikipedia. In 2016 around half of Cochrane Schizophrenia reviews had an obvious 'landing' page directly addressing the topic of the review (30). Then in 2016 we held a one day meeting of student volunteers (medicine and students of applied health sciences), trialists and representatives from Wikipedia and John Wiley Ltd., to plan this trial.(31). The study is a two-arm, parallel, open, randomised controlled trial with a 1:1 allocation ratio.

The aim of this study was to evaluate the impact of seeding relevant Wikipedia pages with evidence from high-grade systematic reviews on information-seeking behaviour.

Eligibility

Inclusion criteria – 'participants'

A Wikipedia page which was clearly relevant to an up-to-date Cochrane Schizophrenia systematic review and that review contained at least one Summary of Findings (SoF) table.

For peer review only

These tables, created within the GradePro(32) system, are succinct summaries of the key outcomes of the review (Box 1).

Exclusion criteria

If a highly relevant Wikipedia page existed but the Cochrane review was out of date with important evidence not incorporated (judgement made by CEA) these pages were not included, and we did not create a new Wikipedia page should one have not existed for an up-to-date review (30). For example, a specialist review such as 'yoga for schizophrenia' would be out of place on a general Wikipedia page 'yoga' and therefore that more general Wikipedia page was ineligible.

Randomisation

Reviews were stratified according to type of intervention (drug or other) and amount of access activity in the year prior to baseline (low or high, according to median split). The latter used Google Analytics' 'pageviews' statistic regarding Cochrane's universally accessible individual review pages(33). The reviews were then allocated to the intervention or control arm by one of the co-authors (AAM) using a computer-generated random number sequence. Allocation was conducted using unique code numbers for each review rather than review title, to avoid risk of selection bias.

Interventions

Experimental group - interventions

Reviews in the intervention group had a referenced table(s) automatically generated by use of SEED(34). This open access software, especially created for this study, uses the original Cochrane review file and re-writes the Cochrane Summary of Findings tables in plain English and generates hyperlink references (to both full subscription review and the universally accessible web summary page) (Figure 1, (35)).

In the design process of our tables we communicated with members of 'Sense about Science'(36) and consulted publications of the Cochrane Effective Practice and Organisation of Care group(37) in order to increase clarity and readability of the evidence in our tables. More details on how we worked to increase readability are described in the protocol(31), as well as our publication of the SEED tool(34). SEED deposits this code in the computer's

For peer review only

memory in seconds. The intervention group's Wikipedia editor (LS, JF) had only to paste this code into the Wikipedia page in the relevant sub-section for the table and hyperlink to appear. This was undertaken across the second week of July 2017.

All content posted in the scope of this trial was sourced from peer-reviewed, systematic reviews published in the Cochrane Library. It complied with WP:MEDRS quality standards for reliable sources in medicine(38). The content posted was intended to improve the encyclopaedia's content, complying with its terms of use. The WP:NOTLAB policy(39) outlines disruptive editing and controversial research. We made an effort to be non-disruptive through discussions with Wikipedia representatives before editing content, as well as using solely verifiable, accessible, and reliable sources. We did not interfere in cases where the re-structuring of Wikipedia articles caused the removal, migration or adaptation of our content, and discuss these cases in our results section.

Control intervention - control

The control group Wikipedia pages did not have a table or reference added – although seven of these pages already had the Cochrane reference employed. This reference was not removed.

Source of data - outcomes

The routine data on full review access is collected by the Cochrane Library's publisher, Wiley. These data, kindly supplied by the Cochrane Office John Wiley, report full text downloads, and Altmetric scores. The latter is a composite weighted measure of the influence of published work online and via social media platforms – in this case composed from monitoring 17 different platforms/news outlets(40) (full list of platforms, and data-by-platform available in data file). The full review is widely accessible(41) but not universally so. Neither is the full review succinct. However, Cochrane Summaries web pages are both universally accessible and succinct and have been awarded for their use of plain English(42). They were monitored using the standard (free) service from Google Analytics(33).

Outcomes

All outcomes were measured at 12 months. There were two outcomes of co-primary interest:

For peer review only

1. The number of visits to the free summary page (All page views)
2. The number of full text downloads

We selected these as the design team(31) felt they represented the best, measurable, most generic indicators of 'more interest' in the evidence as presented in the tables. The first was universally achievable as the webpage for each review is free online. The second – the number of full text downloads – is only possible where this level of access is available. Although coverage of this open service is now considerable (41), this would, nevertheless, mean that some interested readers may not have been registered because of limited access to that outcome. We have no data for this. Secondary outcomes were divided into activity on the free to all summary page, and outcomes relating to activity on the Cochrane Library's full review. More subtle but potentially relevant effects, such as effect on reader behaviour or information comprehension were beyond the scope of the methods used.

Box 1: PICO box

Lists participants, interventions, controls and outcomes

P: Wikipedia pages of direct relevance to up-to-date systematic reviews of the Cochrane Schizophrenia Group
I: Posting the relevant Cochrane review's Summary of Findings table [modified to increase readability] on the target Wikipedia page along with references to the review's web page and full text.
C: Leaving the existing page unmodified
O: Activity on Cochrane web [summary] page specific to that review – thorough use of Google Analytics - and interest in full Cochrane review – through quantification of full text downloads and Altmetric scores of social media activity – though routine data supplied by John Wiley Limited. All at 12 months.

Statistical considerations

The sample size for this study is fixed by the number of eligible Wikipedia pages and Cochrane reviews. From preliminary work we had expected to be able to randomise around 100 pages (30), enabling detection of a between-group standardised difference of 0.57 with 80% power and 5% two-sided alpha. However due to some reviews being too out of date to report on Wikipedia, the actual number available was 70 which permits detection of an effect size of 0.68.

We compared characteristics of the intervention and control arms at baseline using descriptive statistics. For all between-group comparisons, we analysed Wikipedia pages as

For peer review only - http://bmjopen.bmj.com/site/about/guidelines.xhtml

randomised regardless of how long the Wikipedia page held the table. We estimated between-group effects using multivariable linear regression models adjusting for baseline activity, presented with 95% confidence intervals and p-values, and with log-transformation of outcomes as required. For such outcomes, results are presented as ratios of geometric means. Data were analysed using Stata version 15.

Results

All 70 eligible Wikipedia pages relevant to up-to-date Cochrane reviews were randomised, and complete follow up data were available for all (Figure 2).

At baseline Altmetric scores were evenly distributed (Table 2).

Table 2. Baseline altmetric scores

Group	N	Arithmetic Mean	SD	Median	25th centile	75th centile	Min	Max
Control	35	18	30	10	5	19	2	160
Intervention	35	19	24	12	5	25	2	105

During the study 14 of the intervention group's references had additional hyperlinked PubMed IDs added, most probably by Wikipedia's automatic updating service bots. Also, six of the 35 intervention group *tables* were removed after 2 months (3 pages), 5, 8 and 11 months (1 page each) but the *information in the tables* remained within the text as did the hyperlinks (83% of full tables remained 95% CI 67-92%; 100% information remained). As mentioned before, seven of the control pages (20% 95% CI 10-36%) did already have a reference to the relevant Cochrane review. In accordance with WP:NOTLAB policy on minimal disruption to pages (39), and pragmatic trial design in which even 'control' patients may receive some of the experimental treatment if this is in the course of routine care(43), this reference was not removed but no table was added.

One review in the control arm had very high page views (25794, 68x the median for whole sample) but not full text accesses (44) and one review in the intervention arm had very high full text accesses (7407, 18x the median for whole sample (45)).

Although the point estimates for the ratio of geometric means favoured the intervention group for both co-primary outcomes, the confidence intervals were wide and there was no statistical evidence of an effect (Table 3). Results were similar for secondary outcomes, with

For peer review only

the exception of Altmetric score which indicated some evidence of an intervention effect, with 95% confidence interval ranging from 5%-78% increase in geometric mean.

Table 3. Results

Group	N	Arithmetic Mean	SD	Geometric Mean	Adjusted ratio of geometric means	95% confidence interval		p-value
**Co-primary outcomes**								
Full text access								
Control	35	654	721	331	-	-	-	
Intervention	35	994	1448	437	1.30	0.71	2.38	0.39
Page views								
Control	35	1427	4379	318	-	-	-	
Intervention	35	618	656	366	1.14	0.60	2.13	0.69
**Secondary outcomes**								
Altmetric score								
Control	35	19	29	11	-	-	-	
Intervention	35	25	32	15	1.36	1.05	1.78	0.02
Abstract views								
Control	35	364	368	228	-	-	-	
Intervention	35	441	464	271	1.17	0.76	1.81	0.47
Unique page views								
Control	35	1307	4032	290	-	-	-	
Intervention	35	561	596	331	1.13	0.60	2.12	0.70
**Group**	**N**	**Arithmetic Mean**	**SD**		**Adjusted difference in means**	**95% confidence interval**		**p-value**
Time on page (seconds)								
Control	35	165	69	-	-	-	-	
Intervention	35	183	76	-	18.51	-16.06	53.08	0.29

For peer review only

Discussion

This is the first randomised trial of Wikipedia content. Randomisation has been employed before to investigate Wikipedia linguistics(46) but not for the effect of placement of evidence within the page. Our design tried to balance needs of end-users, Wikipedia administrators and editors and methodologists. The intervention was the insertion of an evidence table and references (with hyperlinks) to the source systematic reviews into a highly relevant Wikipedia page. This intervention resulted in no clear, statistically significant,

difference in access to the full review and page views after one year. Although all outcome measures consistently favoured a finding indicating increasing activity on the reviews in the 'intervention' group although only the Altmetric score – a measure of relevant social media activity – reached conventional levels of statistical significance. Inspection of the constituent parts of the composite Altmetric score (please see data file) give no indication that the Wikipedia sub-score is simply causing the elevation in Altmetric ratings. The elevation seemed more linked to micro-blogging sites such as Twitter.

Six tables were deleted at different points across the year out of the 35 inserted into Wikipedia pages. Deletion was undertaken after debate with the Wikipedia user and then the Wikipedia Administrator and is part of the evolution of Wikipedia pages. Administrators have to ensure that this is undertaken in a balanced way taking into account the needs of the readership. Although the tables were deleted, the tables' evidence continued to be reported, as were the hyperlinks. To some readers the tabular format was unacceptable as they felt that tables made the pages "too academic" in appearance. We felt, however, the table was attractive and informative and might encourage interest as well as the seeking of the hyperlink and using it (our primary outcome). Although, after these edits, the hyperlink remained, we think deletion of the table would probably help approximate the results of experimental and control groups. This also illustrates how Wikipedia pages evolve across time. End user feedback is considered and balanced compromises are made. The input to any Wikipedia page, even by respected experts, is not sacrosanct and can be edited in ways that some may not consider advantageous to increasing readership. Working with Wikipedia has the attraction of being dynamic but necessitates commitment, and, for those who feel uncomfortable with their work being edited by unknown others, maintaining Wikipedia evidence could be a less rewarding experience.

The addition of the PubMed IDs broadens the options for gaining additional information for users of the Wikipedia page. However for this trial, again, these additions could have served to narrow any difference between intervention and control. Finally, at the very start of the trial, seven of the *control* pages already had some reference to the Cochrane review. Because of our commitment to minimal disruption of the existing Wikipedia pages and to pragmatism in randomised trials(39,43), we did not feel it right to delete these references

For peer review only

but their presence may also have narrowed the gap between intervention and control groups.

There is little similar literature to contextualise this work. We previously conducted an RCT of Cochrane Schizophrenia review engagement after sending short messages containing review titles or pertinent questions/results relevant to the review via the social media platforms Twitter and Weibo(47). In that study, the primary outcome of increasing views of the review summary page was met, as were several secondary outcomes measuring review engagement (although we did not have data on full text access or Almetric scores). Importantly, the Twitter study measured further review engagement after the relatively few @CochraneSzGroup and Wiebo followers had received a very short fragment (140 characters) of review information. In the current trial, however, we measured engagement after providing the 7,331,024 page viewers (figures for year 10/07/2017-09/07/2018, calculated using Pageview Analysis(48)) to the 70 Wikipedia pages much more evidence (a concise summary-of-findings table). It is possible that the embedded summary-of-findings table may have satiated more readers' appetites for evidence at the time of reading and may have *reduced* the impulse to click out. Also, in the Twitter trial, the 'target' page was one click away. In this Wikipedia trial, the reader had to undertake a minimum of two clicks. Although this difference sounds minimal, it does indicate a considerable commitment of the reader to pursue more information. In this trial, for an outcome to occur, the Wikipedia user had usually to scroll down to find the table, click to expand the drop-down format of the table, seek the reference to that table, and finally click out on one of the hyperlinks. This complex set of actions would, we suggest, indicate high levels of motivation to seek further information and it would seem likely that many users of the Wikipedia pages would have not gone further than the initial page. The Twitter trial suggested a large effect on information-seeking behaviour in a small population, this Wikipedia study did suggest a modest effect – but on a very large population – and in doing this, is important. Many refinements and improvements of this Wikipedia intervention are possible and testable.

Evaluating techniques of dissemination of knowledge is entirely possible and urgent as calls for efficient use of ever-more platforms increase. Much effort may be squandered on attractive but ineffective ideas. This first trial of placement of evidence within Wikipedia supports the need for more evaluative studies of this particular platform. Although only one

For peer review only

secondary outcome reached conventional levels of statistical significance, all outcomes did favour – to some extent – the Wikipedia pages seeded with evidence tables (consistent potential 13-36% increase in activity across all findings). We think this supports the hypothesis that seeding Wikipedia with evidence could be a potent way of encouraging readers to seek more in-depth information on the effects of care. The hit-rate on the 70 very highly specialised Wikipedia pages was over 500K/month. If even half were the activity of robotic automated systems(49) that still leaves considerable activity from interested people. How best to seed good evidence into Wikipedia, how best to communicate with this readership, how to use images and infographics, and how to work with Wikipedia to best advantage of all, all are possible to evaluate in future research.

Conclusions

The care Wikipedia invests in the contents of health pages is considerable and the 'live' 'crowd-sourced' and adjudicated peer reviewing of pages is impressive. The outcomes we were able to use are likely to be only the tip of the 'activity iceberg' and placing evidence within Wikipedia seems likely to raise the profile of – in this case – the effects of care.

For peer review only

Trial organisation

The trial was sponsored by the Nottinghamshire Healthcare NHS Foundation Trust. We had no clear reasons to establish a Data Monitoring Committee or a Steering Committee.

Trial registration

IRCT2017070330407N2

Acknowledgements

The research team is grateful to Julie Wood and the Communications and External Affairs team of the Cochrane Collaboration who gave permission for use of the access code for the summaries pages via Google Analytics.

Thanks also to Joanne Thomas and Max Goldman (Sense about Science - senseaboutscience.org) who helped with the language within the tables.

We also wish to thank James Heilman (Wikipedian, The Wikipedia Open Textbook of Medicine) for his ongoing support in seeding Wikipedia with good evidence.

Data sharing

All data relevant to the study are available in a public, open access repository - Permanent URL: https://doi.org/10.17605/OSF.IO/K2SP4

Protocol

https://doi.org/10.22541/au.149926363.33383675

Copyright

The Corresponding Author has the right to grant on behalf of all authors and does grant on behalf of all authors, a worldwide licence to the Publishers and its licensees in perpetuity, in all forms, formats and media (whether known now or created in the future), to i) publish, reproduce, distribute, display and store the Contribution, ii) translate the Contribution into other languages, create adaptations, reprints, include within collections and create summaries, extracts and/or, abstracts of the Contribution, iii) create any other derivative work(s) based on the Contribution, iv) to exploit all subsidiary rights in the Contribution, v) the inclusion of electronic links from the Contribution to third party material where-ever it may be located; and, vi) licence any third party to do any or all of the above.

For peer review only

Competing interest statement

There are no competing interests for any author. All authors have completed the Unified Competing Interest form (available on request from the corresponding author) and declare: no support from any organisation for the submitted work [or describe if any]; no financial relationships with any organisations that might have an interest in the submitted work in the previous three years [or describe if any], no other relationships or activities that could appear to have influenced the submitted work [or describe if any].

All posts and edits were undertaken by the user Lena08041993, with any conflicts of interest and affiliation of this account with the Cochrane Schizophrenia Group clearly declared on the user's talk page (https://en.wikipedia.org/wiki/User:Lena08041993).

Douglas Taylor is affiliated to the Wikimedia Foundation.

Transparency declaration

The lead author (the manuscript's guarantor) affirms that the manuscript is an honest, accurate, and transparent account of the study being reported; that no important aspects of the study have been omitted; and that any discrepancies from the study as planned (and, if relevant, registered) have been explained.

Details of ethical approval (or a statement that it was not required).

This study employed inanimate Cochrane systematic review-generated Wikipedia tables as participants and collected routine data from electronic systems for outcomes. We enquired of the local Ethics Committee and were advised that ethical approval is not required. As such, this study is a prototype for ethical randomised interventions in Wikipedia.

Details of funding

This study was funded by the UK Economic and Social Research Council (ESRC) Impact *Accelerator Fund* (£7.5K) with intramural support from the University of Nottingham.

Statement of independence of researchers from funders

The researchers have no relationship with the ESRC.

Patient and public involvement statement

For peer review only

We did not have patient involvement. However, we did have the involvement of the public. The protocol for this trial (31) was created by a group of Wikipedia users – medical and informatics students. In March 2017 we organised a one day meeting to support consultation meeting with students for this trial. This was funded by ESRC (£2.5K of the total described above specifically for this meeting).

The meeting, led by methodologists, also had attendance of representatives of the publisher of the Cochrane Library (John Wiley Ltd.) and of Wikipedia. However, the primary purpose of the day was to get consultation on how the trial should be undertaken from the perspective of one end-user group of Wikipedia – the students. They have continued to be involved in the drafting and writing of the protocol, the conduct of the trial and this final draft report.

Trial registration details (registry and number)

This appears at the end of the abstract (including hyperlink). Recognising that registration is important to help consideration by the major journals, we sought this registration early on – at protocol stage. We were informed that we could not register, as we were not randomising human beings. Because Cochrane Schizophrenia's Information Specialist is from Iran, he knew that some local registries do not apply this rule and that key local registries also are uploaded into the international systems – and this includes the registry from Iran – hence why this study is registered there.

Patient consent

Not applicable

Contributorship statement

Authors

Clive E Adams - envisioned and led the project and gained [modest] funding for it and contributed – in substantial measure - to the planning, conduct and reporting of the trial.

Alan A Montgomery - helped led the project and undertook the analyses and contributed – in substantial measure - to the planning, conduct and reporting of the trial.

Tony Aburrow - contributed – in substantial measure - to the planning, conduct and reporting of the trial and greatly assisted data acquisition from John Wiley.

Sophie Bloomfield - contributed – in substantial measure - to the planning, conduct and reporting of the trial.

Paul M Briley - contributed – in substantial measure - to the planning, conduct and reporting of the trial.

Ebun Carew - contributed – in substantial measure - to the planning, conduct and reporting of the trial.

Suravi Chatterjee-Woolman - contributed – in substantial measure - to the planning, conduct and reporting of the trial.

Ghalia Feddah - contributed – in substantial measure - to the planning, conduct and reporting of the trial.

Johannes Friedel - contributed – in substantial measure - to the planning, conduct and reporting of the trial and undertook software design (SEED) and particularly helped with data acquisition from Google Analytics.

Josh Gibbard - contributed – in substantial measure - to the planning, conduct and reporting of the trial.

Euan Haynes - contributed – in substantial measure - to the planning, conduct and reporting of the trial.

Mohsin Hussein - contributed – in substantial measure - to the planning, conduct and reporting of the trial.

Mahesh Jayaram - contributed – in substantial measure - to the planning, conduct and reporting of the trial.

Samuel D Naylor - contributed – in substantial measure - to the planning, conduct and reporting of the trial.

Luke Perry - contributed – in substantial measure - to the planning, conduct and reporting of the trial.

Lena Schmidt - contributed – in substantial measure - to the planning, conduct and reporting of the trial and undertook software design (SEED) and particularly helped with data acquisition from Google Analytics.

Umer Siddique - contributed – in substantial measure - to the planning, conduct and reporting of the trial.

Ayla S Tabaksert - contributed – in substantial measure - to the planning, conduct and reporting of the trial.

Douglas Taylor - contributed – in substantial measure - to the planning, conduct and reporting of the trial and provided continual help with the Wikipedia perspective.

Aarti Velani - contributed – in substantial measure - to the planning, conduct and reporting of the trial.

Douglas White - contributed – in substantial measure - to the planning, conduct and reporting of the trial.

Jun Xia - contributed – in substantial measure - to the planning, conduct and reporting of the trial.

Contributions

Julie Wood and the Communications and External Affairs team of the Cochrane Collaboration gave permission for use of the access code for the summaries pages via Google Analytics.

Joanne Thomas and Max Goldman (Sense about Science - senseaboutscience.org) helped with the language to be used within the tables.

James Heilman (Wikipedian, The Wikipedia Open Textbook of Medicine) supported seeding Wikipedia with good evidence.

For peer review only

References

1. Wikipedia:About. In: Wikipedia [Internet]. 2019 [cited 2019 Nov 13]. Available from: https://en.wikipedia.org/w/index.php?title=Wikipedia:About&oldid=922393621

2. Wikipedia:Size of Wikipedia. In: Wikipedia [Internet]. 2018 [cited 2018 Dec 3]. Available from: https://en.wikipedia.org/w/index.php?title=Wikipedia:Size_of_Wikipedia&oldid=871450371

3. Monica, erson, Hitlin P, Atkinson M. Wikipedia at 15: Millions of readers in scores of languages [Internet]. Pew Research Center. [cited 2018 Dec 3]. Available from: http://www.pewresearch.org/fact-tank/2016/01/14/wikipedia-at-15/

4. Wikimedia Statistics - All wikis [Internet]. [cited 2018 Dec 3]. Available from: https://stats.wikimedia.org/v2/#/all-projects

5. Alexa Top 500 Global Sites [Internet]. [cited 2017 Apr 10]. Available from: http://www.alexa.com/topsites

6. Heilman J, Bonert M, Beards G, Thomas B, Vondracek A, Grover S, et al. Wikipedia: A Key Tool for Global Public Health Promotion. J Med Internet Res [Internet]. 2011 Mar [cited 2016 Aug 4];13(1). Available from: http://www.jmir.org/2011/1/e14/?trendmd-shared=1

7. Health information on Wikipedia. In: Wikipedia [Internet]. 2019 [cited 2019 Nov 13]. Available from: https://en.wikipedia.org/w/index.php?title=Health_information_on_Wikipedia&oldid=924170161

8. Makovsky. Online Health Research Eclipsing Patient-Doctor Conversations [Internet]. 2013 [cited 2016 Apr 8]. Available from: http://www.makovsky.com/news/online-health-research-eclipsing-patient-doctor-conversations/

9. Laurent M, Vickers T. Seeking health information online: does Wikipedia matter? J Am Med Inform Assoc. 2009 Jul;16(4):471–9.

10. Heilman J, West A. Wikipedia and Medicine: Quantifying Readership, Editors, and the Significance of Natural Language. J Med Internet Res [Internet]. 2015 Mar [cited 2016 Aug 4];17(3). Available from: https://www.ncbi.nlm.nih.gov/pmc/articles/PMC4376174/

11. Hughes B, Joshi I, Lemonde H, Wareham J. Junior physician's use of Web 2.0 for information seeking and medical education: a qualitative study. Int J Med Inf. 2009 Oct;78(10):645–55.

12. Hughes B, Wareham J, Joshi I. Doctors' online information needs, cognitive search strategies, and judgments of information quality and cognitive authority: How predictive judgments introduce bias into cognitive search models. J Assoc Inf Sci Technol. 2010 Mar;61(3):433–52.

13. Rajagopalan M, Khanna V, Leiter Y, Stott M, Showalter T, Dicker A, et al. Patient-Oriented Cancer Information on the Internet: A Comparison of Wikipedia and a Professionally Maintained Database. J Oncol Pract. 2011 Sep;7(5):319–23.

14. Hasty RT, Garbalosa RC, Barbato VA, Valdes PJ, Powers DW, Hernandez E, et al. Wikipedia vs peer-reviewed medical literature for information about the 10 most costly medical conditions. J Am Osteopath Assoc. 2014 May;114(5):368–73.

For peer review only

15. Leithner A, Maurer-Ertl W, Glehr M, Friesenbichler J, Leithner K, Windhager R. Wikipedia and osteosarcoma: a trustworthy patients' information? J Am Med Inform Assoc JAMIA. 2010 Aug;17(4):373–4.

16. Azer SA, AlSwaidan NM, Alshwairikh LA, AlShammari JM. Accuracy and readability of cardiovascular entries on Wikipedia: are they reliable learning resources for medical students? BMJ Open. 2015;5(10):e008187.

17. Phillips J, Lam C, Palmisano L. Analysis of the accuracy and readability of herbal supplement information on Wikipedia. J Am Pharm Assoc JAPhA. 2014 Aug;54(4):406–14.

18. Azer SA. Evaluation of gastroenterology and hepatology articles on Wikipedia: are they suitable as learning resources for medical students? Eur J Gastroenterol Hepatol. 2014 Feb;26(2):155–63.

19. Reavley NJ, Mackinnon AJ, Morgan AJ, Alvarez-Jimenez M, Hetrick SE, Killackey E, et al. Quality of information sources about mental disorders: a comparison of Wikipedia with centrally controlled web and printed sources. Psychol Med. 2012 Aug;42(8):1753–62.

20. Thomas GR, Eng L, de Wolff JF, Grover SC. An evaluation of Wikipedia as a resource for patient education in nephrology. Semin Dial. 2013 Apr;26(2):159–63.

21. Aldairy T, Laverick S, McIntyre GT. Orthognathic surgery: is patient information on the Internet valid? Eur J Orthod. 2012 Aug;34(4):466–9.

22. Reilly T, Jackson W, Berger V, Candelario D. Accuracy and completeness of drug information in Wikipedia medication monographs. J Am Pharm Assoc JAPhA. 2017 Apr;57(2):193-196.e1.

23. Kräenbring J, Monzon Penza T, Gutmann J, Muehlich S, Zolk O, Wojnowski L, et al. Accuracy and completeness of drug information in Wikipedia: a comparison with standard textbooks of pharmacology. PloS One. 2014;9(9):e106930.

24. Clauson KA, Polen HH, Boulos MNK, Dzenowagis JH. Scope, completeness, and accuracy of drug information in Wikipedia. Ann Pharmacother. 2008 Dec;42(12):1814–21.

25. Azer SA. Is Wikipedia a reliable learning resource for medical students? Evaluating respiratory topics. Adv Physiol Educ. 2015 Mar;39(1):5–14.

26. The Cochrane Collaboration | Working together to provide the best evidence for health care [Internet]. [cited 2011 May 16]. Available from: http://www.cochrane.org/

27. Cochrane Schizophrenia Group. Cochrane Schizophrenia Group Website [Internet]. 2013 [cited 2013 Sep 25]. Available from: http://szg.cochrane.org/

28. Wikipedia:Cochrane Collaboration/Cochrane UK/About. In: Wikipedia [Internet]. 2017 [cited 2019 Nov 13]. Available from: https://en.wikipedia.org/w/index.php?title=Wikipedia:Cochrane_Collaboration/Cochrane_UK/About&oldid=798337875

29. Home | OpenPrescribing [Internet]. [cited 2019 Nov 15]. Available from: https://openprescribing.net/

For peer review only

30. White D, Adams CE. What proportion of Cochrane Reviews are Wiki-compatible? | Cochrane Community [Internet]. [cited 2017 Apr 10]. Available from: /news/what-proportion-cochrane-reviews-are-wiki-compatible

31. Adams CE, Montgomery AA, Aburrow A, Bloomfield S, Briley P, Carew E, et al. The effects of Wikipedia referencing: a protocol for a randomised trial. Authorea Prepr [Internet]. 2017 Jul 5 [cited 2017 Oct 12]; Available from: https://www.authorea.com/users/163403/articles/185228-the-effects-of-wikipedia-referencing-a-protocol-for-a-randomised-trial

32. GRADEpro | GDT [Internet]. [cited 2017 Apr 10]. Available from: https://gradepro.org/

33. Google Team. Google Analytics [Internet]. 2014 [cited 2014 Jul 2]. Available from: http://www.google.com/analytics/

34. Schmidt L, Friedel J, Adams CE. SEED: a tool for disseminating systematic review data into Wikipedia. Syst Rev. 2017 Oct 17;6(1):206.

35. Quetiapine. In: Wikipedia [Internet]. 2019 [cited 2019 Jun 11]. Available from: https://en.wikipedia.org/w/index.php?title=Quetiapine&oldid=894522053

36. Sense about Science – Because evidence matters [Internet]. [cited 2017 Jul 10]. Available from: http://senseaboutscience.org/

37. Cochrane Effective Practice and Organisation of Care (EPOC) Group [Internet]. [cited 2019 Nov 14]. Available from: /about-us

38. Wikipedia:Identifying reliable sources (medicine). In: Wikipedia [Internet]. 2019 [cited 2019 Nov 14]. Available from: https://en.wikipedia.org/w/index.php?title=Wikipedia:Identifying_reliable_sources_(medicine)&oldid=925910307

39. Wikipedia:What Wikipedia is not. In: Wikipedia [Internet]. 2019 [cited 2019 Nov 14]. Available from: https://en.wikipedia.org/w/index.php?title=Wikipedia:What_Wikipedia_is_not&oldid=926025458

40. The Importance of Altmetrics: A Primer | Wiley [Internet]. [cited 2018 Dec 12]. Available from: https://hub.wiley.com/community/exchanges/discover/blog/2016/06/01/the-importance-of-altmetrics-a-primer

41. List of countries eligible for free one-click access to the Cochrane Library in 2017 [Internet]. [cited 2019 Nov 14]. Available from: https://community.cochrane.org/news/list-countries-eligible-free-one-click-access-cochrane-library-2017

42. Cochrane Summaries Receives Award [Internet]. [cited 2019 Nov 14]. Available from: /news/cochrane-summaries-receives-award

43. Thorpe KE, Zwarenstein M, Oxman AD, Treweek S, Furberg CD, Altman DG, et al. A pragmatic-explanatory continuum indicator summary (PRECIS): a tool to help trial designers. J Clin Epidemiol. 2009 May;62(5):464–75.

For peer review only

44. Skelton M, Khokhar WA, Thacker SP. Treatments for delusional disorder. Cochrane Database Syst Rev. 2015 May 22;(5):CD009785.

45. Soares-Weiser K, Maayan N, Bergman H, Davenport C, Kirkham AJ, Grabowski S, et al. First rank symptoms for schizophrenia. Cochrane Database Syst Rev. 2015;1:CD010653.

46. Thompson N, Hanley D. Science Is Shaped by Wikipedia: Evidence From a Randomized Control Trial [Internet]. Rochester, NY: Social Science Research Network; 2018 Feb [cited 2019 Nov 13]. Report No.: ID 3039505. Available from: https://papers.ssrn.com/abstract=3039505

47. Adams CE, Jayaram M, Bodart AYM, Sampson S, Zhao S, Montgomery AA. Tweeting links to Cochrane Schizophrenia Group reviews: a randomised controlled trial. BMJ Open. 2016;6(3):e010509.

48. Pageviews Analysis [Internet]. [cited 2019 Nov 19]. Available from: https://tools.wmflabs.org/pageviews/?project=en.wikipedia.org&platform=all-access&agent=user&range=latest-20&pages=Cat|Dog

49. Malicious bot traffic climbs 9.5 percent in 2017, says report [Internet]. SC Media. 2018 [cited 2019 May 21]. Available from: https://www.scmagazine.com/home/research/malicious-bot-traffic-climbs-9-5-percent-in-2017-says-report/

Figure Legend

Figure 1: Sample of embedded table

Figure 2. CONSORT flow diagram

For peer review only

Paliperidone palmitate long-acting injection compared to risperidone for schizophrenia[2]			
When flexibly dosed every four weeks, paliperidone palmitate appears comparable in efficacy and tolerability to risperidone. In short-term studies, paliperidone palmitate – the longer-acting injection – has a similar adverse effect profile to related compounds such as risperidone by mouth. No difference was found in the high rate of reported adverse sexual outcomes and paliperidone palmitate is associated with an increase in serum prolactin.[2]			
[hide] Outcome	Findings in words	Findings in numbers	Quality of evidence
**Global state: No clinically important change**			
No 30% improvement on PANSS score. Follow-up: 13-53 weeks	There is no clear difference between people given paliperidone palmitate and those receiving risperidone for this outcome. These findings are based on data of low quality.	RR 1.03 (0.93 to 1.14)	Low
**Relapse**			
Recurrence of psychotic symptoms. Follow up: 13-53 weeks	There is no clear difference between people given paliperidone palmitate and those receiving risperidone for the outcome of 'relapse'. Data supporting this finding are based on moderate quality evidence.	RR 1.23 (0.98 to 1.53)	Moderate
**Leaving the study early**			
- For any reason. Follow up: 13-53 weeks	Paliperidone palmitate causes little or no increase to the chance of leaving the study.	RR 1.12 (1 to 1.25)	High

Figure 1: Example of embedded table

221x165mm (96 x 96 DPI)

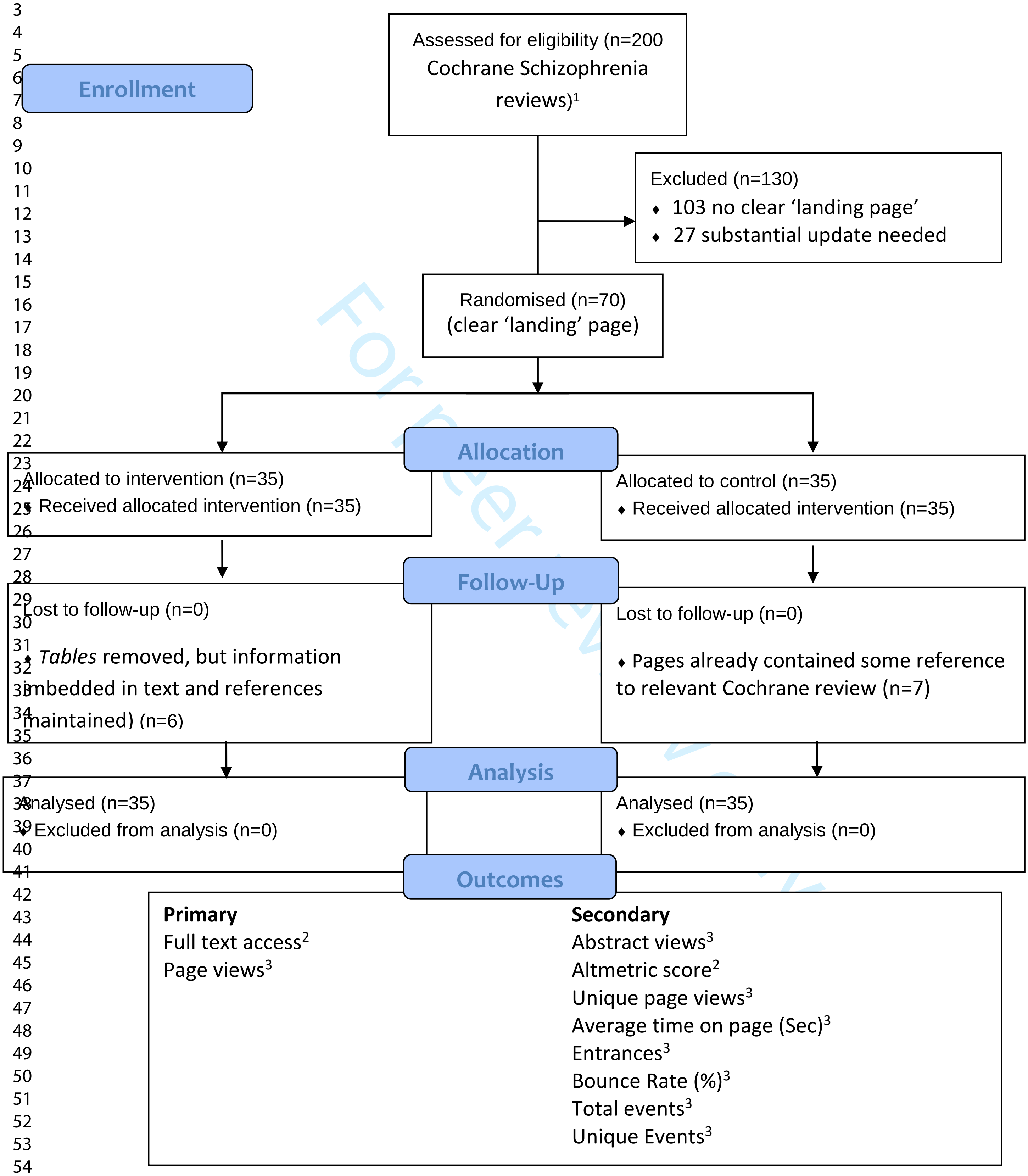

1. 2016 **Data source** 2. John Wiley Ltd 3. Google Analytics

For peer review only - http://bmjopen.bmj.com/site/about/guidelines.xhtml

CONSORT checklist of items for reporting pragmatic trials

Section	Item	Standard CONSORT description	Extension for pragmatic trials	Line
Title and abstract	1	How participants were allocated to interventions (eg, "random allocation," "randomised," or "randomly assigned")		CONSORT1
**Introduction**				
Background	2	Scientific background and explanation of rationale	Describe the health or health service problem that the intervention is intended to address and other interventions that may commonly be aimed at this problem	CONSORT2
**Methods**				
Participants	3	Eligibility criteria for participants; settings and locations where the data were collected	Eligibility criteria should be explicitly framed to show the degree to which they include typical participants and/or, where applicable, typical providers (eg, nurses), institutions (eg, hospitals), communities (or localities eg, towns) and settings of care (eg, different healthcare financing systems)	CONSORT3
Interventions	4	Precise details of the interventions intended for each group and how and when they were actually administered	Describe extra resources added to (or resources removed from) usual settings in order to implement intervention. Indicate if efforts were made to standardise the intervention or if the intervention and its delivery were allowed to vary between participants, practitioners, or study sites	CONSORT4

For peer review only

Section	Item	Standard CONSORT description	Extension for pragmatic trials	Line
			Describe the comparator in similar detail to the intervention	
Objectives	5	Specific objectives and hypotheses		CONSORT5
Outcomes	6	Clearly defined primary and secondary outcome measures and, when applicable, any methods used to enhance the quality of measurements (eg, multiple observations, training of assessors)	Explain why the chosen outcomes and, when relevant, the length of follow-up are considered important to those who will use the results of the trial	CONSORT6
Sample size	7	How sample size was determined; explanation of any interim analyses and stopping rules when applicable	If calculated using the smallest difference considered important by the target decision maker audience (the minimally important difference) then report where this difference was obtained	CONSORT7
Randomisation—sequence generation	8	Method used to generate the random allocation sequence, including details of any restriction (eg, blocking, stratification)		CONSORT8
Randomisation—allocation concealment	9	Method used to implement the random allocation sequence (eg, numbered containers or central telephone), clarifying whether the sequence was concealed until interventions were assigned		CONSORT8
Randomisation—implementation	10	Who generated the allocation sequence, who enrolled participants, and who assigned participants to their groups		CONSORT8
Blinding (masking)	11	Whether participants, those administering the interventions, and those assessing the outcomes were blinded to group assignment	If blinding was not done, or was not possible, explain why	CONSORT9

For peer review only

Section	Item	Standard CONSORT description	Extension for pragmatic trials	Line
Statistical methods	12	Statistical methods used to compare groups for primary outcomes; methods for additional analyses, such as subgroup analyses and adjusted analyses		CONSORT10
**Results**				
Participant flow	13	Flow of participants through each stage (a diagram is strongly recommended)—specifically, for each group, report the numbers of participants randomly assigned, receiving intended treatment, completing the study protocol, and analysed for the primary outcome; describe deviations from planned study protocol, together with reasons	The number of participants or units approached to take part in the trial, the number which were eligible, and reasons for non-participation should be reported	Figure 2
Recruitment	14	Dates defining the periods of recruitment and follow-up		CONSORT11 CONSORT12
Baseline data	15	Baseline demographic and clinical characteristics of each group		Table 2
Numbers analysed	16	Number of participants (denominator) in each group included in each analysis and whether analysis was by "intention-to-treat"; state the results in absolute numbers when feasible (eg, 10/20, not 50%)		Figure 2
Outcomes and estimation	17	For each primary and secondary outcome, a summary of results for each group and the estimated effect size and its precision (eg, 95% CI)		Table 3

For peer review only

Section	Item	Standard CONSORT description	Extension for pragmatic trials	Line
Ancillary analyses	18	Address multiplicity by reporting any other analyses performed, including subgroup analyses and adjusted analyses, indicating which are prespecified and which are exploratory		N/A
Adverse events	19	All important adverse events or side effects in each intervention group		N/A
**Discussion**				
Interpretation	20	Interpretation of the results, taking into account study hypotheses, sources of potential bias or imprecision, and the dangers associated with multiplicity of analyses and outcomes		CONSORT13
Generalisability	21	Generalisability (external validity) of the trial findings	Describe key aspects of the setting which determined the trial results. Discuss possible differences in other settings where clinical traditions, health service organisation, staffing, or resources may vary from those of the trial	CONSORT14
Overall evidence	22	General interpretation of the results in the context of current evidence		CONSORT14

Cite as: *Zwarenstein M, Treweek S, Gagnier JJ, Altman DG, Tunis S, Haynes B, Oxman AD, Moher D for the CONSORT and Pragmatic Trials in Healthcare (Practihc) group. Improving the reporting of pragmatic trials: an extension of the CONSORT statement. BMJ 2008; 337;a2390.*

For peer review only

BMJ Open

Adding evidence of the effects of treatments into relevant Wikipedia pages: a randomised trial.

Journal:	*BMJ Open*
Manuscript ID	bmjopen-2019-033655.R2
Article Type:	Research
Date Submitted by the Author:	03-Jan-2020
Complete List of Authors:	Adams, Clive; University of Nottingham, Division of Psychiatry and Applied Psychology<br>Montgomery, Alan; University of Nottingham, Nottingham Clinical Trials Unit<br>Aburrow, Tony; John Wiley and Sons, Limited<br>Bloomfield, Sophie; East Kent Hospitals University NHS Foundation Trust<br>Briley, Paul; University of Nottingham, Division of Psychiatry and Applied Psychology<br>Carew, Ebun; Nottingham University Hospitals Healthcare NHS Trust<br>Chatterjee-Woolman, Suravi; Sherwood Forest Hospitals NHS Foundation Trust<br>Feddah, Ghalia; Sherwood Forest Hospitals NHS Foundation Trust<br>Friedel, Johannes; University of Aalen<br>Gibbard, Josh; Morecambe Bay Hospitals NHS Trust<br>Haynes, Euan; Gateshead Health NHS Foundation Trust<br>Hussein, Mohsin; University Hospitals of Leicester NHS Trust<br>Jayaram, Mahesh; University of Melbourne, Psychaitry<br>Naylor, Samuel; Sherwood Forest Hospitals NHS Foundation Trust<br>Perry, Luke; Royal Melbourne Hospital<br>Schmidt, Lena; University of Bristol Faculty of Health Sciences<br>Siddique, Umer; Barking Havering and Redbridge Hospitals NHS Trust<br>Tabaksert, Ayla; Northumbria Healthcare NHS Foundation Trust<br>Taylor, Douglas; Wikimedia Foundation Inc<br>Velani, Aarti; Nottingham University Hospitals NHS Trust<br>White, Douglas; Epsom and Saint Helier University Hospitals NHS Trust Epsom Hospital<br>Xia, Jun; Nottingham China Health Institute, The University of Nottingham Ningbo
**Primary Subject Heading**:	Health informatics
Secondary Subject Heading:	Medical education and training, Medical publishing and peer review, Mental health
Keywords:	MEDICAL EDUCATION & TRAINING, Schizophrenia & psychotic disorders < PSYCHIATRY, World Wide Web technology < BIOTECHNOLOGY & BIOINFORMATICS

For peer review only - http://bmjopen.bmj.com/site/about/guidelines.xhtml

I, the Submitting Author has the right to grant and does grant on behalf of all authors of the Work (as defined in the below author licence), an exclusive licence and/or a non-exclusive licence for contributions from authors who are: i) UK Crown employees; ii) where BMJ has agreed a CC-BY licence shall apply, and/or iii) in accordance with the terms applicable for US Federal Government officers or employees acting as part of their official duties; on a worldwide, perpetual, irrevocable, royalty-free basis to BMJ Publishing Group Ltd ("BMJ") its licensees and where the relevant Journal is co-owned by BMJ to the co-owners of the Journal, to publish the Work in this journal and any other BMJ products and to exploit all rights, as set out in our licence.

The Submitting Author accepts and understands that any supply made under these terms is made by BMJ to the Submitting Author unless you are acting as an employee on behalf of your employer or a postgraduate student of an affiliated institution which is paying any applicable article publishing charge ("APC") for Open Access articles. Where the Submitting Author wishes to make the Work available on an Open Access basis (and intends to pay the relevant APC), the terms of reuse of such Open Access shall be governed by a Creative Commons licence – details of these licences and which Creative Commons licence will apply to this Work are set out in our licence referred to above.

Other than as permitted in any relevant BMJ Author's Self Archiving Policies, I confirm this Work has not been accepted for publication elsewhere, is not being considered for publication elsewhere and does not duplicate material already published. I confirm all authors consent to publication of this Work and authorise the granting of this licence.

For peer review only

Title: Adding evidence of the effects of treatments into relevant Wikipedia pages: a randomised trial.

Authors:

Clive E Adams, Alan A Montgomery, Tony Aburrow, Sophie Bloomfield, Paul M Briley, Ebun Carew, Suravi Chatterjee-Woolman, Ghalia Feddah, Johannes Friedel, Josh Gibbard, Euan Haynes, Mohsin Hussein, Mahesh Jayaram, Samuel D Naylor, Luke Perry, Lena Schmidt, Umer Siddique, Ayla S Tabaksert, Douglas Taylor, Aarti Velani, Douglas White, Jun Xia

Contact:

Professor Clive E Adams

Chair Mental Health Services Research, Co-ordinating Editor, Cochrane Schizophrenia

Institute of Mental Health

Jubilee Campus

University of Nottingham Innovation Park

Triumph Road

Nottingham

NG7 2TU

Phone: 0115 823 1294

Fax: 0115 823 1294

Email: clive.adams@nottingham.ac.uk

Co-authors:

Alan A Montgomery, Nottingham Clinical Trials Unit, University of Nottingham, Nottingham, UK,
Tony Aburrow, John Wiley Ltd., Chichester, UK,

Sophie Bloomfield, East Kent Hospitals University Foundation Trust, Ashford, UK

Paul M Briley, Institute of Mental Health, University of Nottingham, Nottingham, UK

Ebun Carew, Nottingham University Hospitals Healthcare NHS Trust, Nottingham, UK

Suravi Chatterjee-Woolman, Sherwood Forest Hospitals NHS Foundation Trust, Sutton in Ashfield, UK

Ghalia Feddah, Sherwood Forest Hospitals NHS Foundation Trust, Sutton in Ashfield, UK

Johannes Friedel, University of Aalen, Aalen, Germany

Josh Gibbard, Morecambe Bay Hospitals NHS Trust, Lancaster, UK

Euan Haynes, Gateshead Health, NHS Foundation Trust, Gateshead, UK

Mohsin Hussein, Leicester Evington Centre, Leicester, UK

Mahesh Jayaram, Department of Psychiatry, University of Melbourne, Australia

Samuel D Naylor, Sherwood Forest Hospitals NHS Foundation Trust, Sutton in Ashfield, UK

Luke Perry, Royal Melbourne Hospital, Parkville, Australia

Lena Schmidt, Hochschule Furtwangen University, Furtwangen, Germany; University of Bristol, Bristol, UK

Umer Siddique, Barking, Havering and Redbridge Hospitals NHS Trust, London, UK

Ayla S Tabaksert, Northumbria NHS Foundation Trust, Newcastle upon Tyne, UK

Douglas Taylor, Wikimedia UK, Loughborough, UK

Aarti Velani, Nottingham University Hospitals Healthcare NHS Trust, Nottingham, UK

Douglas White, Epsom and St Helier University Hospitals NHS Trust, London, UK

Jun Xia, Nottingham China Health Institute, The University of Nottingham Ningbo, Ningbo, China

Key words

Medical education

Health Informatics

Psychiatry

Randomized controlled trial

Word count 3207

For peer review only

Abstract

Objectives

To investigate the effects of adding high-grade quantitative evidence of outcomes of treatments into relevant Wikipedia pages on further information-seeking behaviour by use of routinely collected data.

Setting

Wikipedia, Cochrane summary pages and the Cochrane Library.

Design

Randomised trial.

Participants

Wikipedia pages which were highly relevant to up-to-date Cochrane Schizophrenia systematic reviews that contained a Summary of Findings table.

Interventions

Eligible Wikipedia pages in the intervention group were seeded with tables of best evidence of the effects of care and hyperlinks to the source Cochrane review. Eligible Wikipedia pages in the control group were left unchanged.

Main outcome measures

Routinely collected data on access to the full text and summary web-page (after 12 months).

Results

We randomised 70 Wikipedia pages (100% follow up). Six of the 35 Wikipedia pages in the intervention group had the tabular format deleted during the study but all pages continued to report the same data within the text. There was no evidence of effect on either of the co-primary outcomes: full text access adjusted ratio of geometric means 1.30, 95% CI 0.71 to 2.38; page views 1.14, 95% CI 0.6 to 2.13. Results were similar for all other outcomes, with exception of Altmetric score for which there was some evidence of clear effect (1.36, 95% CI 1.05 to 1.78.

For peer review only

Conclusions

The pursuit of fair balance within Wikipedia health care pages is impressive and its reach unsurpassed. For every person who sought and clicked the reference on the 'intervention' Wikipedia page to seek more information (the primary outcome), many more are likely to have been informed by the page alone. Enriching Wikipedia content is, potentially, a powerful way to improve health literacy and it is possible to test the effects of seeding pages with evidence. This trial should be replicated, expanded and developed.

[Abstract 298 words]

Trial registration

[Prospectively registered 20/07/2017; IRCT2017070330407N2]

Strengths and limitations of this study

- First randomised trial of placement of evidence within Wikipedia pages
- Use of routine data to allow 100% follow up
- Open editing of Wikipedia pages – both intervention and control pages - by the Wikipedia community served to minimise difference between groups.
- Outcomes necessitated unusual levels of interest and commitment on the part of the Wikipedia page reader
- Small study in highly specialised area of heath care

For peer review only

Background

Wikipedia is a free-content online encyclopaedia containing articles on a vast range of topics(1). At present there are over 5.7m articles, 46 million pages in the English language(2). Since its creation in 2001, Wikipedia has expanded to attract over 27m registered users(3) with 16 billion pages views per month(4). This made Wikipedia the 5th most popular site on the internet in 2017(5).

Wikipedia is openly-editable. This means that any one of these users can access *and edit* the majority of articles. Wikipedia policy states, however, that all information presented in pages must be "verifiable against a published reliable source"(1). Therefore, all pages aim to contain references for the information they provide. To prevent the risk of pages being devalued with misinformation Wikipedia has various quality control measures. These include; a 'watchlist' to notify editors when a page has been edited, a published list of recent changes that editors can access to review, automated computer scripts, page protection on more controversial pages, edit filters on certain pages and blocking any editors who repeatedly damage the value of the page (6). On top of this, Wikipedia has a team of administrators. They are editors who have been given access to additional tools on their account. These include the ability to block/unblock accounts, edit fully protected pages and delete/undelete pages. There are 1,194 administrators on the English language Wikipedia (as of December 2018)(2).

Wikipedia contains many pages relating to healthcare. In 2014 the English language version was estimated to contain 25,000 articles on health-related topics, while across all languages there are 155,000 articles containing 950,000 references(7). These are often accessed via search engine results with one survey suggesting that around 22% of healthcare-related online searches direct to Wikipedia pages(8,9). In 2013 health pages on Wikipedia received 4.8 billion views, making it one of the most used means for accessing health information globally(10). When use of Wikipedia is studied in medical students and doctors, it is clear that it is becoming an increasingly popular resource(11,12). This is, perhaps, enhanced by Wikipedia being entirely free of charge – including data download charges in low and middle income countries. In this context there is criticism that as Wikipedia is openly editable, the information it contains may be unreliable. Some evidence suggests, however, that there is no difference in accuracy when Wikipedia is compared to other professionally maintained

For peer review only

medical databases(13) although opinions differ by subspecialty, depend on the ‘target’ readership and vary across time (Table 1).

Table 1: Selection of studies of Wikipedia’s value to different readerships by medical subspecialty

Sub-specialty (reference)	Date	Assessing for suitability for….	Conclusion
**10 most costly conditions (14)**	2014	General readership	Most Wikipedia articles representing the 10 most costly medical conditions [….] contain many errors when checked against standard peer-reviewed sources. Caution should be used […..]
**Cancer – general (13)**	2011	Patients	Wiki resource had similar accuracy and depth as the professionally edited database
**Cancer – osteoscarcoma (15)**	2010	Patients	[…] the quality of osteosarcoma-related information found in the English Wikipedia is good but inferior to the patient information provided by the NCI.
**Cardiovascular (16)**	2015	Medical students	Wikipedia entries are not aimed at a medical audience and should not be used as a substitute to recommended medical resources. Course designers and students should be aware that Wikipedia entries on cardiovascular diseases lack accuracy, predominantly due to errors of omission.
**Complementary medicine (17)**	2014	General readership	Patients and health professionals should not rely solely on Wikipedia for information on these herbal supplements when treatment decisions are being made.
**Gastro – hepatology (18)**	2014	Medical students	…..not good source of evidence
**Mental health (19)**	2012	General readership	The quality of information on depression and schizophrenia on Wikipedia is generally as good as, or better than, that provided by centrally controlled

			websites, Encyclopaedia Britannica and a psychiatry textbook.
**Nephrology (20)**	2013	Patients	Fairly reliable medical resource
**Orthognathic surgery (21)**	2012	Patients	Maximum […] score[ings in comparison to other online sources] were Wikipedia
**Pharmacology (22)**	2017	Doctors	Wikipedia lacks the accuracy and completeness of standard clinical references and should not be a routine part of clinical decision making.
**Pharmacology (23)**	2014	Medical students	… Wikipedia is an accurate and comprehensive source of drug-related information for undergraduate medical education.
**Pharmacology (24)**	2008	Patients	Wikipedia has a more narrow scope, is less complete, and has more errors of omission than the comparator database. Wikipedia may be a useful point of engagement for consumers, but is not authoritative and should only be a supplemental source of drug information.
**Respiratory medicine (25)**	2015	Medical students	Most articles had knowledge deficiencies, were not accurate, and were not suitable for medical students as learning resources.

The Cochrane Collaboration(26) is a non-for profit NGO producing, and maintaining systematic reviews of health care published within the *Cochrane Library (*by John Wiley Ltd.). The Collaboration is made up of subgroups and Cochrane Schizophrenia produces and updates high quality systematic reviews and meta-analyses relevant to people with schizophrenia and related psychotic conditions(27). In 2004 a group called WikiProject Medicine was started with the aim of creating and managing medical articles on Wikipedia. This group allows discussion and collaboration on these articles to improve the quality of the information presented(6). In 2014 a formalised partnership between Wikipedia and Cochrane was created, aiming to “transform the quality and content of health evidence available online”(28). This involves incorporating Cochrane’s evidence into Wikipedia articles and improving the information’s accuracy and reliability.

For peer review only

Whilst increasing accessibility of highest grade maintained health care information seems a laudable aim, objective quantification of the effects of this effort has not been undertaken. This paper reports a collaboratively designed pragmatic randomised trial of adding evidence of the effects of care to Wikipedia health pages on the routinely collected indicators of readers' interest.

Aims

To evaluate the effects of enriching Wikipedia content with summary tables from level 1 evidence on the effects of care.

Methods

In preliminary work, we tested stability of target pages in Wikipedia. Adding an evidence-table to four Wikipedia pages (trifluoperazine – a less used antipsychotic e.g 3529 ± 198 prescriptions/month - figures are for 2018, NHS England(29); chlorpromazine – a old widely-used antipsychotic drug 22386 ±803 prescriptions/month; palperidone – a expensive new antipsychotic drug 853 ± 34 prescriptions/month, and one important talking therapy - cognitive behavioural therapy). These four pages all remained stable over a 12 month period(2015). Further work investigated what proportion of the topics of Cochrane Schizophrenia reviews already had a highly specific page in Wikipedia. In 2016 around half of Cochrane Schizophrenia reviews had an obvious 'landing' page directly addressing the topic of the review (30). Then in 2016 we held a one day meeting of student volunteers (medicine and students of applied health sciences), trialists and representatives from Wikipedia and John Wiley Ltd., to plan this trial.(31). The study is a two-arm, parallel, open, randomised controlled trial with a 1:1 allocation ratio.

The aim of this study was to evaluate the impact of seeding relevant Wikipedia pages with evidence from high-grade systematic reviews on information-seeking behaviour.

Eligibility

Inclusion criteria – 'participants'

A Wikipedia page which was clearly relevant to an up-to-date Cochrane Schizophrenia systematic review and that review contained at least one Summary of Findings (SoF) table.

For peer review only

These tables, created within the GradePro(32) system, are succinct summaries of the key outcomes of the review (Box 1).

Exclusion criteria

If a highly relevant Wikipedia page existed but the Cochrane review was out of date with important evidence not incorporated (judgement made by CEA) these pages were not included, and we did not create a new Wikipedia page should one have not existed for an up-to-date review (30). For example, a specialist review such as ‘yoga for schizophrenia’ would be out of place on a general Wikipedia page ‘yoga’ and therefore that more general Wikipedia page was ineligible.

Randomisation

Reviews were stratified according to type of intervention (drug or other) and amount of access activity in the year prior to baseline (low or high, according to median split). The latter used Google Analytics’ ‘pageviews’ statistic regarding Cochrane’s universally accessible individual review pages(33). The reviews were then allocated to the intervention or control arm by one of the co-authors (AAM) using a computer-generated random number sequence. Allocation was conducted using unique code numbers for each review rather than review title, to avoid risk of selection bias.

Interventions

Experimental group - interventions

Reviews in the intervention group had a referenced table(s) automatically generated by use of SEED(34). This open access software, especially created for this study, uses the original Cochrane review file and re-writes the Cochrane Summary of Findings tables in plain English and generates hyperlink references (to both full subscription review and the universally accessible web summary page) (Figure 1, (35)).

In the design process of our tables we communicated with members of ‘Sense about Science’(36) and consulted publications of the Cochrane Effective Practice and Organisation of Care group(37) in order to increase clarity and readability of the evidence in our tables. More details on how we worked to increase readability are described in the protocol(31), as well as our publication of the SEED tool(34). SEED deposits this code in the computer’s

For peer review only

memory in seconds. The intervention group's Wikipedia editor (LS, JF) had only to paste this code into the Wikipedia page in the relevant sub-section for the table and hyperlink to appear. This was undertaken across the second week of July 2017.

All content posted in the scope of this trial was sourced from peer-reviewed, systematic reviews published in the Cochrane Library. It complied with WP:MEDRS quality standards for reliable sources in medicine(38). The content posted was intended to improve the encyclopaedia's content, complying with its terms of use. The WP:NOTLAB policy(39) outlines disruptive editing and controversial research. We made an effort to be non-disruptive through discussions with Wikipedia representatives before editing content, as well as using solely verifiable, accessible, and reliable sources. We did not interfere in cases where the re-structuring of Wikipedia articles caused the removal, migration or adaptation of our content, and discuss these cases in our results section.

Control intervention - control

The control group Wikipedia pages did not have a table or reference added – although seven of these pages already had the Cochrane reference employed. This reference was not removed.

Source of data - outcomes

The routine data on full review access is collected by the Cochrane Library's publisher, Wiley. These data, kindly supplied by the Cochrane Office John Wiley, report full text downloads, and Altmetric scores. The latter is a composite weighted measure of the influence of published work online and via social media platforms – in this case composed from monitoring 17 different platforms/news outlets(40) (full list of platforms, and data-by-platform available in data file at https://doi.org/10.17605/OSF.IO/K2SP4). The full review is widely accessible(41) but not universally so. Neither is the full review succinct. However, Cochrane Summaries web pages are both universally accessible and succinct and have been awarded for their use of plain English(42). They were monitored using the standard (free) service from Google Analytics(33).

Outcomes

All outcomes were measured at 12 months. There were two outcomes of co-primary interest:

For peer review only

1. The number of visits to the free summary page (All page views)
2. The number of full text downloads

We selected these as the design team(31) felt they represented the best, measurable, most generic indicators of 'more interest' in the evidence as presented in the tables. The first was universally achievable as the webpage for each review is free online. The second – the number of full text downloads – is only possible where this level of access is available. Although coverage of this open service is now considerable (41), this would, nevertheless, mean that some interested readers may not have been registered because of limited access to that outcome. We have no data for this. Secondary outcomes were divided into activity on the free to all summary page, and outcomes relating to activity on the Cochrane Library's full review. More subtle but potentially relevant effects, such as effect on reader behaviour or information comprehension were beyond the scope of the methods used.

Box 1: PICO box

Lists participants, interventions, controls and outcomes

P: Wikipedia pages of direct relevance to up-to-date systematic reviews of the Cochrane Schizophrenia Group
I: Posting the relevant Cochrane review's Summary of Findings table [modified to increase readability] on the target Wikipedia page along with references to the review's web page and full text.
C: Leaving the existing page unmodified
O: Activity on Cochrane web [summary] page specific to that review – thorough use of Google Analytics - and interest in full Cochrane review – through quantification of full text downloads and Altmetric scores of social media activity – though routine data supplied by John Wiley Limited. All at 12 months.

Statistical considerations

The sample size for this study is fixed by the number of eligible Wikipedia pages and Cochrane reviews. From preliminary work we had expected to be able to randomise around 100 pages (30), enabling detection of a between-group standardised difference of 0.57 with 80% power and 5% two-sided alpha. However due to some reviews being too out of date to report on Wikipedia, the actual number available was 70 which permits detection of an effect size of 0.68.

We compared characteristics of the intervention and control arms at baseline using descriptive statistics. For all between-group comparisons, we analysed Wikipedia pages as

randomised regardless of how long the Wikipedia page held the table. We estimated between-group effects using multivariable linear regression models adjusting for baseline activity, presented with 95% confidence intervals and p-values, and with log-transformation of outcomes as required. For such outcomes, results are presented as ratios of geometric means. Data were analysed using Stata version 15.

Results

All 70 eligible Wikipedia pages relevant to up-to-date Cochrane reviews were randomised, and complete follow up data were available for all (Figure 2).

At baseline Altmetric scores were evenly distributed (Table 2).

Table 2. Baseline altmetric scores

Group	N	Arithmetic Mean	SD	Median	25th centile	75th centile	Min	Max
Control	35	18	30	10	5	19	2	160
Intervention	35	19	24	12	5	25	2	105

During the study 14 of the intervention group's references had additional hyperlinked PubMed IDs added, most probably by Wikipedia's automatic updating service bots. Also, six of the 35 intervention group *tables* were removed after 2 months (3 pages), 5, 8 and 11 months (1 page each) but the *information in the tables* remained within the text as did the hyperlinks (83% of full tables remained 95% CI 67-92%; 100% information remained). As mentioned before, seven of the control pages (20% 95% CI 10-36%) did already have a reference to the relevant Cochrane review. In accordance with WP:NOTLAB policy on minimal disruption to pages (39), and pragmatic trial design in which even 'control' patients may receive some of the experimental treatment if this is in the course of routine care(43), this reference was not removed but no table was added.

One review in the control arm had very high page views (25794, 68x the median for whole sample) but not full text accesses (44) and one review in the intervention arm had very high full text accesses (7407, 18x the median for whole sample (45)).

Although the point estimates for the ratio of geometric means favoured the intervention group for both co-primary outcomes, the confidence intervals were wide and there was no statistical evidence of an effect (Table 3). Results were similar for secondary outcomes, with

For peer review only

the exception of Altmetric score which indicated some evidence of an intervention effect, with 95% confidence interval ranging from 5%-78% increase in geometric mean.

Table 3. Results

Group	N	Arithmetic Mean	SD	Geometric Mean	Adjusted ratio of geometric means	95% confidence interval		p-value
**Co-primary outcomes**								
Full text access								
Control	35	654	721	331	-	-	-	
Intervention	35	994	1448	437	1.30	0.71	2.38	0.39
Page views								
Control	35	1427	4379	318	-	-	-	
Intervention	35	618	656	366	1.14	0.60	2.13	0.69
**Secondary outcomes**								
Altmetric score								
Control	35	19	29	11	-	-	-	
Intervention	35	25	32	15	1.36	1.05	1.78	0.02
Abstract views								
Control	35	364	368	228	-	-	-	
Intervention	35	441	464	271	1.17	0.76	1.81	0.47
Unique page views								
Control	35	1307	4032	290	-	-	-	
Intervention	35	561	596	331	1.13	0.60	2.12	0.70
**Group**	**N**	**Arithmetic Mean**	**SD**		**Adjusted difference in means**	**95% confidence interval**		**p-value**
Time on page (seconds)								
Control	35	165	69	-	-	-	-	
Intervention	35	183	76	-	18.51	-16.06	53.08	0.29

Discussion

This is the first randomised trial of Wikipedia content. Randomisation has been employed before to investigate Wikipedia linguistics(46) but not for the effect of placement of evidence within the page. Our design tried to balance needs of end-users, Wikipedia administrators and editors and methodologists. The intervention was the insertion of an evidence table and references (with hyperlinks) to the source systematic reviews into a highly relevant Wikipedia page. This intervention resulted in no clear, statistically significant,

difference in access to the full review and page views after one year. Although all outcome measures consistently favoured a finding indicating increasing activity on the reviews in the 'intervention' group although only the Altmetric score – a measure of relevant social media activity – reached conventional levels of statistical significance. Inspection of the constituent parts of the composite Altmetric score (please see data file at https://doi.org/10.17605/OSF.IO/K2SP4) give no indication that the Wikipedia sub-score is simply causing the elevation in Altmetric ratings. The elevation seemed more linked to micro-blogging sites such as Twitter.

Six tables were deleted at different points across the year out of the 35 inserted into Wikipedia pages. Deletion was undertaken after debate with the Wikipedia user and then the Wikipedia Administrator and is part of the evolution of Wikipedia pages. Administrators have to ensure that this is undertaken in a balanced way taking into account the needs of the readership. Although the tables were deleted, the tables' evidence continued to be reported, as were the hyperlinks. To some readers the tabular format was unacceptable as they felt that tables made the pages "too academic" in appearance. We felt, however, the table was attractive and informative and might encourage interest as well as the seeking of the hyperlink and using it (our primary outcome). Although, after these edits, the hyperlink remained, we think deletion of the table would probably help approximate the results of experimental and control groups. This also illustrates how Wikipedia pages evolve across time. End user feedback is considered and balanced compromises are made. The input to any Wikipedia page, even by respected experts, is not sacrosanct and can be edited in ways that some may not consider advantageous to increasing readership. Working with Wikipedia has the attraction of being dynamic but necessitates commitment, and, for those who feel uncomfortable with their work being edited by unknown others, maintaining Wikipedia evidence could be a less rewarding experience.

The addition of the PubMed IDs broadens the options for gaining additional information for users of the Wikipedia page. However for this trial, again, these additions could have served to narrow any difference between intervention and control. Finally, at the very start of the trial, seven of the *control* pages already had some reference to the Cochrane review. Because of our commitment to minimal disruption of the existing Wikipedia pages and to pragmatism in randomised trials(39,43), we did not feel it right to delete these references

For peer review only

but their presence may also have narrowed the gap between intervention and control groups.

There is little similar literature to contextualise this work. We previously conducted an RCT of Cochrane Schizophrenia review engagement after sending short messages containing review titles or pertinent questions/results relevant to the review via the social media platforms Twitter and Weibo(47). In that study, the primary outcome of increasing views of the review summary page was met, as were several secondary outcomes measuring review engagement (although we did not have data on full text access or Almetric scores). Importantly, the Twitter study measured further review engagement after the relatively few @CochraneSzGroup and Wiebo followers had received a very short fragment (140 characters) of review information. In the current trial, however, we measured engagement after providing the 7,331,024 page viewers (figures for year 10/07/2017-09/07/2018, calculated using Pageview Analysis(48)) to the 70 Wikipedia pages much more evidence (a concise summary-of-findings table). It is possible that the embedded summary-of-findings table may have satiated more readers' appetites for evidence at the time of reading and may have *reduced* the impulse to click out. Also, in the Twitter trial, the 'target' page was one click away. In this Wikipedia trial, the reader had to undertake a minimum of two clicks. Although this difference sounds minimal, it does indicate a considerable commitment of the reader to pursue more information. In this trial, for an outcome to occur, the Wikipedia user had usually to scroll down to find the table, click to expand the drop-down format of the table, seek the reference to that table, and finally click out on one of the hyperlinks. This complex set of actions would, we suggest, indicate high levels of motivation to seek further information and it would seem likely that many users of the Wikipedia pages would have not gone further than the initial page. The Twitter trial suggested a large effect on information-seeking behaviour in a small population, this Wikipedia study did suggest a modest effect – but on a very large population – and in doing this, is important. Many refinements and improvements of this Wikipedia intervention are possible and testable.

Evaluating techniques of dissemination of knowledge is entirely possible and urgent as calls for efficient use of ever-more platforms increase. Much effort may be squandered on attractive but ineffective ideas. This first trial of placement of evidence within Wikipedia supports the need for more evaluative studies of this particular platform. Although only one

For peer review only

secondary outcome reached conventional levels of statistical significance, all outcomes did favour – to some extent – the Wikipedia pages seeded with evidence tables (consistent potential 13-36% increase in activity across all findings). We think this supports the hypothesis that seeding Wikipedia with evidence could be a potent way of encouraging readers to seek more in-depth information on the effects of care. The hit-rate on the 70 very highly specialised Wikipedia pages was over 500K/month. If even half were the activity of robotic automated systems(49) that still leaves considerable activity from interested people. How best to seed good evidence into Wikipedia, how best to communicate with this readership, how to use images and infographics, and how to work with Wikipedia to best advantage of all, all are possible to evaluate in future research.

Conclusions

The care Wikipedia invests in the contents of health pages is considerable and the 'live' 'crowd-sourced' and adjudicated peer reviewing of pages is impressive. The outcomes we were able to use are likely to be only the tip of an 'activity iceberg'. For every person who sought and clicked the reference on the 'intervention' Wikipedia page to seek more information (the primary outcome), many more are likely to have been informed by the page alone. Enriching Wikipedia content is, potentially, a powerful way to improve health literacy and it is possible to test the effects of seeding pages with evidence. This trial should be replicated, expanded and developed.

For peer review only

Trial organisation

The trial was sponsored by the Nottinghamshire Healthcare NHS Foundation Trust. We had no clear reasons to establish a Data Monitoring Committee or a Steering Committee.

Trial registration

IRCT2017070330407N2

Acknowledgements

The research team is grateful to Julie Wood and the Communications and External Affairs team of the Cochrane Collaboration who gave permission for use of the access code for the summaries pages via Google Analytics.

Thanks also to Joanne Thomas and Max Goldman (Sense about Science - senseaboutscience.org) who helped with the language within the tables.

We also wish to thank James Heilman (Wikipedian, The Wikipedia Open Textbook of Medicine) for his ongoing support in seeding Wikipedia with good evidence.

Data sharing

All data relevant to the study are available in a public, open access repository - Permanent URL: https://doi.org/10.17605/OSF.IO/K2SP4

Protocol

https://doi.org/10.22541/au.149926363.33383675

Copyright

The Corresponding Author has the right to grant on behalf of all authors and does grant on behalf of all authors, a worldwide licence to the Publishers and its licensees in perpetuity, in all forms, formats and media (whether known now or created in the future), to i) publish, reproduce, distribute, display and store the Contribution, ii) translate the Contribution into other languages, create adaptations, reprints, include within collections and create summaries, extracts and/or, abstracts of the Contribution, iii) create any other derivative work(s) based on the Contribution, iv) to exploit all subsidiary rights in the Contribution, v) the inclusion of electronic links from the Contribution to third party material where-ever it may be located; and, vi) licence any third party to do any or all of the above.

For peer review only

Competing interest statement

There are no competing interests for any author. All authors have completed the Unified Competing Interest form (available on request from the corresponding author) and declare: no support from any organisation for the submitted work [or describe if any]; no financial relationships with any organisations that might have an interest in the submitted work in the previous three years [or describe if any], no other relationships or activities that could appear to have influenced the submitted work [or describe if any].

All posts and edits were undertaken by the user Lena08041993, with any conflicts of interest and affiliation of this account with the Cochrane Schizophrenia Group clearly declared on the user's talk page (https://en.wikipedia.org/wiki/User:Lena08041993).

Douglas Taylor is affiliated to the Wikimedia Foundation.

Transparency declaration

The lead author (the manuscript's guarantor) affirms that the manuscript is an honest, accurate, and transparent account of the study being reported; that no important aspects of the study have been omitted; and that any discrepancies from the study as planned (and, if relevant, registered) have been explained.

Details of ethical approval (or a statement that it was not required).

This study employed inanimate Cochrane systematic review-generated Wikipedia tables as participants and collected routine data from electronic systems for outcomes. We enquired of the local Ethics Committee and were advised that ethical approval is not required. As such, this study is a prototype for ethical randomised interventions in Wikipedia.

Details of funding

This study was funded by the UK Economic and Social Research Council (ESRC) Impact *Accelerator Fund* (£7.5K) with intramural support from the University of Nottingham.

Statement of independence of researchers from funders

The researchers have no relationship with the ESRC.

Patient and public involvement statement

For peer review only

We did not have patient involvement. However, we did have the involvement of the public. The protocol for this trial (31) was created by a group of Wikipedia users – medical and informatics students. In March 2017 we organised a one day meeting to support consultation meeting with students for this trial. This was funded by ESRC (£2.5K of the total described above specifically for this meeting).

The meeting, led by methodologists, also had attendance of representatives of the publisher of the Cochrane Library (John Wiley Ltd.) and of Wikipedia. However, the primary purpose of the day was to get consultation on how the trial should be undertaken from the perspective of one end-user group of Wikipedia – the students. They have continued to be involved in the drafting and writing of the protocol, the conduct of the trial and this final draft report.

Trial registration details (registry and number)

This appears at the end of the abstract (including hyperlink). Recognising that registration is important to help consideration by the major journals, we sought this registration early on – at protocol stage. We were informed that we could not register, as we were not randomising human beings. Because Cochrane Schizophrenia's Information Specialist is from Iran, he knew that some local registries do not apply this rule and that key local registries also are uploaded into the international systems – and this includes the registry from Iran – hence why this study is registered there.

Patient consent

Not applicable

Contributorship statement

Authors

Clive E Adams - envisioned and led the project and gained [modest] funding for it and contributed – in substantial measure - to the planning, conduct and reporting of the trial.

Alan A Montgomery - helped led the project and undertook the analyses and contributed – in substantial measure - to the planning, conduct and reporting of the trial.

Tony Aburrow - contributed – in substantial measure - to the planning, conduct and reporting of the trial and greatly assisted data acquisition from John Wiley.

Sophie Bloomfield - contributed – in substantial measure - to the planning, conduct and reporting of the trial.

Paul M Briley - contributed – in substantial measure - to the planning, conduct and reporting of the trial.

Ebun Carew - contributed – in substantial measure - to the planning, conduct and reporting of the trial.

Suravi Chatterjee-Woolman - contributed – in substantial measure - to the planning, conduct and reporting of the trial.

Ghalia Feddah - contributed – in substantial measure - to the planning, conduct and reporting of the trial.

Johannes Friedel - contributed – in substantial measure - to the planning, conduct and reporting of the trial and undertook software design (SEED) and particularly helped with data acquisition from Google Analytics.

Josh Gibbard - contributed – in substantial measure - to the planning, conduct and reporting of the trial.

Euan Haynes - contributed – in substantial measure - to the planning, conduct and reporting of the trial.

Mohsin Hussein - contributed – in substantial measure - to the planning, conduct and reporting of the trial.

Mahesh Jayaram - contributed – in substantial measure - to the planning, conduct and reporting of the trial.

Samuel D Naylor - contributed – in substantial measure - to the planning, conduct and reporting of the trial.

Luke Perry - contributed – in substantial measure - to the planning, conduct and reporting of the trial.

Lena Schmidt - contributed – in substantial measure - to the planning, conduct and reporting of the trial and undertook software design (SEED) and particularly helped with data acquisition from Google Analytics.

Umer Siddique - contributed – in substantial measure - to the planning, conduct and reporting of the trial.

Ayla S Tabaksert - contributed – in substantial measure - to the planning, conduct and reporting of the trial.

Douglas Taylor - contributed – in substantial measure - to the planning, conduct and reporting of the trial and provided continual help with the Wikipedia perspective.

Aarti Velani - contributed – in substantial measure - to the planning, conduct and reporting of the trial.

Douglas White - contributed – in substantial measure - to the planning, conduct and reporting of the trial.

Jun Xia - contributed – in substantial measure - to the planning, conduct and reporting of the trial.

Contributions

Julie Wood and the Communications and External Affairs team of the Cochrane Collaboration gave permission for use of the access code for the summaries pages via Google Analytics.

Joanne Thomas and Max Goldman (Sense about Science - senseaboutscience.org) helped with the language to be used within the tables.

James Heilman (Wikipedian, The Wikipedia Open Textbook of Medicine) supported seeding Wikipedia with good evidence.

References

1. Wikipedia:About. In: Wikipedia [Internet]. 2019 [cited 2019 Nov 13]. Available from: https://en.wikipedia.org/w/index.php?title=Wikipedia:About&oldid=922393621

2. Wikipedia:Size of Wikipedia. In: Wikipedia [Internet]. 2018 [cited 2018 Dec 3]. Available from: https://en.wikipedia.org/w/index.php?title=Wikipedia:Size_of_Wikipedia&oldid=871450371

3. Monica, erson, Hitlin P, Atkinson M. Wikipedia at 15: Millions of readers in scores of languages [Internet]. Pew Research Center. [cited 2018 Dec 3]. Available from: http://www.pewresearch.org/fact-tank/2016/01/14/wikipedia-at-15/

4. Wikimedia Statistics - All wikis [Internet]. [cited 2018 Dec 3]. Available from: https://stats.wikimedia.org/v2/#/all-projects

5. Alexa Top 500 Global Sites [Internet]. [cited 2017 Apr 10]. Available from: http://www.alexa.com/topsites

6. Heilman J, Bonert M, Beards G, Thomas B, Vondracek A, Grover S, et al. Wikipedia: A Key Tool for Global Public Health Promotion. J Med Internet Res [Internet]. 2011 Mar [cited 2016 Aug 4];13(1). Available from: http://www.jmir.org/2011/1/e14/?trendmd-shared=1

7. Health information on Wikipedia. In: Wikipedia [Internet]. 2019 [cited 2019 Nov 13]. Available from: https://en.wikipedia.org/w/index.php?title=Health_information_on_Wikipedia&oldid=924170161

8. Makovsky. Online Health Research Eclipsing Patient-Doctor Conversations [Internet]. 2013 [cited 2016 Apr 8]. Available from: http://www.makovsky.com/news/online-health-research-eclipsing-patient-doctor-conversations/

9. Laurent M, Vickers T. Seeking health information online: does Wikipedia matter? J Am Med Inform Assoc. 2009 Jul;16(4):471–9.

10. Heilman J, West A. Wikipedia and Medicine: Quantifying Readership, Editors, and the Significance of Natural Language. J Med Internet Res [Internet]. 2015 Mar [cited 2016 Aug 4];17(3). Available from: https://www.ncbi.nlm.nih.gov/pmc/articles/PMC4376174/

11. Hughes B, Joshi I, Lemonde H, Wareham J. Junior physician's use of Web 2.0 for information seeking and medical education: a qualitative study. Int J Med Inf. 2009 Oct;78(10):645–55.

12. Hughes B, Wareham J, Joshi I. Doctors' online information needs, cognitive search strategies, and judgments of information quality and cognitive authority: How predictive judgments introduce bias into cognitive search models. J Assoc Inf Sci Technol. 2010 Mar;61(3):433–52.

13. Rajagopalan M, Khanna V, Leiter Y, Stott M, Showalter T, Dicker A, et al. Patient-Oriented Cancer Information on the Internet: A Comparison of Wikipedia and a Professionally Maintained Database. J Oncol Pract. 2011 Sep;7(5):319–23.

14. Hasty RT, Garbalosa RC, Barbato VA, Valdes PJ, Powers DW, Hernandez E, et al. Wikipedia vs peer-reviewed medical literature for information about the 10 most costly medical conditions. J Am Osteopath Assoc. 2014 May;114(5):368–73.

For peer review only

15. Leithner A, Maurer-Ertl W, Glehr M, Friesenbichler J, Leithner K, Windhager R. Wikipedia and osteosarcoma: a trustworthy patients' information? J Am Med Inform Assoc JAMIA. 2010 Aug;17(4):373–4.

16. Azer SA, AlSwaidan NM, Alshwairikh LA, AlShammari JM. Accuracy and readability of cardiovascular entries on Wikipedia: are they reliable learning resources for medical students? BMJ Open. 2015;5(10):e008187.

17. Phillips J, Lam C, Palmisano L. Analysis of the accuracy and readability of herbal supplement information on Wikipedia. J Am Pharm Assoc JAPhA. 2014 Aug;54(4):406–14.

18. Azer SA. Evaluation of gastroenterology and hepatology articles on Wikipedia: are they suitable as learning resources for medical students? Eur J Gastroenterol Hepatol. 2014 Feb;26(2):155–63.

19. Reavley NJ, Mackinnon AJ, Morgan AJ, Alvarez-Jimenez M, Hetrick SE, Killackey E, et al. Quality of information sources about mental disorders: a comparison of Wikipedia with centrally controlled web and printed sources. Psychol Med. 2012 Aug;42(8):1753–62.

20. Thomas GR, Eng L, de Wolff JF, Grover SC. An evaluation of Wikipedia as a resource for patient education in nephrology. Semin Dial. 2013 Apr;26(2):159–63.

21. Aldairy T, Laverick S, McIntyre GT. Orthognathic surgery: is patient information on the Internet valid? Eur J Orthod. 2012 Aug;34(4):466–9.

22. Reilly T, Jackson W, Berger V, Candelario D. Accuracy and completeness of drug information in Wikipedia medication monographs. J Am Pharm Assoc JAPhA. 2017 Apr;57(2):193-196.e1.

23. Kräenbring J, Monzon Penza T, Gutmann J, Muehlich S, Zolk O, Wojnowski L, et al. Accuracy and completeness of drug information in Wikipedia: a comparison with standard textbooks of pharmacology. PloS One. 2014;9(9):e106930.

24. Clauson KA, Polen HH, Boulos MNK, Dzenowagis JH. Scope, completeness, and accuracy of drug information in Wikipedia. Ann Pharmacother. 2008 Dec;42(12):1814–21.

25. Azer SA. Is Wikipedia a reliable learning resource for medical students? Evaluating respiratory topics. Adv Physiol Educ. 2015 Mar;39(1):5–14.

26. The Cochrane Collaboration | Working together to provide the best evidence for health care [Internet]. [cited 2011 May 16]. Available from: http://www.cochrane.org/

27. Cochrane Schizophrenia Group. Cochrane Schizophrenia Group Website [Internet]. 2013 [cited 2013 Sep 25]. Available from: http://szg.cochrane.org/

28. Wikipedia:Cochrane Collaboration/Cochrane UK/About. In: Wikipedia [Internet]. 2017 [cited 2019 Nov 13]. Available from: https://en.wikipedia.org/w/index.php?title=Wikipedia:Cochrane_Collaboration/Cochrane_UK/About&oldid=798337875

29. Home | OpenPrescribing [Internet]. [cited 2019 Nov 15]. Available from: https://openprescribing.net/

For peer review only

30. White D, Adams CE. What proportion of Cochrane Reviews are Wiki-compatible? | Cochrane Community [Internet]. [cited 2017 Apr 10]. Available from: /news/what-proportion-cochrane-reviews-are-wiki-compatible

31. Adams CE, Montgomery AA, Aburrow A, Bloomfield S, Briley P, Carew E, et al. The effects of Wikipedia referencing: a protocol for a randomised trial. Authorea Prepr [Internet]. 2017 Jul 5 [cited 2017 Oct 12]; Available from: https://www.authorea.com/users/163403/articles/185228-the-effects-of-wikipedia-referencing-a-protocol-for-a-randomised-trial

32. GRADEpro | GDT [Internet]. [cited 2017 Apr 10]. Available from: https://gradepro.org/

33. Google Team. Google Analytics [Internet]. 2014 [cited 2014 Jul 2]. Available from: http://www.google.com/analytics/

34. Schmidt L, Friedel J, Adams CE. SEED: a tool for disseminating systematic review data into Wikipedia. Syst Rev. 2017 Oct 17;6(1):206.

35. Quetiapine. In: Wikipedia [Internet]. 2019 [cited 2019 Jun 11]. Available from: https://en.wikipedia.org/w/index.php?title=Quetiapine&oldid=894522053

36. Sense about Science – Because evidence matters [Internet]. [cited 2017 Jul 10]. Available from: http://senseaboutscience.org/

37. Cochrane Effective Practice and Organisation of Care (EPOC) Group [Internet]. [cited 2019 Nov 14]. Available from: /about-us

38. Wikipedia:Identifying reliable sources (medicine). In: Wikipedia [Internet]. 2019 [cited 2019 Nov 14]. Available from: https://en.wikipedia.org/w/index.php?title=Wikipedia:Identifying_reliable_sources_(medicine)&oldid=925910307

39. Wikipedia:What Wikipedia is not. In: Wikipedia [Internet]. 2019 [cited 2019 Nov 14]. Available from: https://en.wikipedia.org/w/index.php?title=Wikipedia:What_Wikipedia_is_not&oldid=926025458

40. The Importance of Altmetrics: A Primer | Wiley [Internet]. [cited 2018 Dec 12]. Available from: https://hub.wiley.com/community/exchanges/discover/blog/2016/06/01/the-importance-of-altmetrics-a-primer

41. List of countries eligible for free one-click access to the Cochrane Library in 2017 [Internet]. [cited 2019 Nov 14]. Available from: https://community.cochrane.org/news/list-countries-eligible-free-one-click-access-cochrane-library-2017

42. Cochrane Summaries Receives Award [Internet]. [cited 2019 Nov 14]. Available from: /news/cochrane-summaries-receives-award

43. Thorpe KE, Zwarenstein M, Oxman AD, Treweek S, Furberg CD, Altman DG, et al. A pragmatic-explanatory continuum indicator summary (PRECIS): a tool to help trial designers. J Clin Epidemiol. 2009 May;62(5):464–75.

For peer review only

44. Skelton M, Khokhar WA, Thacker SP. Treatments for delusional disorder. Cochrane Database Syst Rev. 2015 May 22;(5):CD009785.

45. Soares-Weiser K, Maayan N, Bergman H, Davenport C, Kirkham AJ, Grabowski S, et al. First rank symptoms for schizophrenia. Cochrane Database Syst Rev. 2015;1:CD010653.

46. Thompson N, Hanley D. Science Is Shaped by Wikipedia: Evidence From a Randomized Control Trial [Internet]. Rochester, NY: Social Science Research Network; 2018 Feb [cited 2019 Nov 13]. Report No.: ID 3039505. Available from: https://papers.ssrn.com/abstract=3039505

47. Adams CE, Jayaram M, Bodart AYM, Sampson S, Zhao S, Montgomery AA. Tweeting links to Cochrane Schizophrenia Group reviews: a randomised controlled trial. BMJ Open. 2016;6(3):e010509.

48. Pageviews Analysis [Internet]. [cited 2019 Nov 19]. Available from: https://tools.wmflabs.org/pageviews/?project=en.wikipedia.org&platform=all-access&agent=user&range=latest-20&pages=Cat|Dog

49. Malicious bot traffic climbs 9.5 percent in 2017, says report [Internet]. SC Media. 2018 [cited 2019 May 21]. Available from: https://www.scmagazine.com/home/research/malicious-bot-traffic-climbs-9-5-percent-in-2017-says-report/

Figure Legend

Figure 1: Sample of embedded table

Figure 2. CONSORT flow diagram

For peer review only

**Paliperidone palmitate long-acting injection compared to risperidone for schizophrenia**[2]			
When flexibly dosed every four weeks, paliperidone palmitate appears comparable in efficacy and tolerability to risperidone. In short-term studies, paliperidone palmitate – the longer-acting injection – has a similar adverse effect profile to related compounds such as risperidone by mouth. No difference was found in the high rate of reported adverse sexual outcomes and paliperidone palmitate is associated with an increase in serum prolactin.[2]			
[hide] **Outcome**	**Findings in words**	**Findings in numbers**	**Quality of evidence**
**Global state: No clinically important change**			
No 30% improvement on PANSS score. Follow-up: 13-53 weeks	There is no clear difference between people given paliperidone palmitate and those receiving risperidone for this outcome. These findings are based on data of low quality.	RR 1.03 (0.93 to 1.14)	Low
**Relapse**			
Recurrence of psychotic symptoms. Follow up: 13-53 weeks	There is no clear difference between people given paliperidone palmitate and those receiving risperidone for the outcome of 'relapse'. Data supporting this finding are based on moderate quality evidence.	RR 1.23 (0.98 to 1.53)	Moderate
**Leaving the study early**			
- For any reason. Follow up: 13-53 weeks	Paliperidone palmitate causes little or no increase to the chance of leaving the study.	RR 1.12 (1 to 1.25)	High

Figure 1: Example of embedded table

221x165mm (96 x 96 DPI)

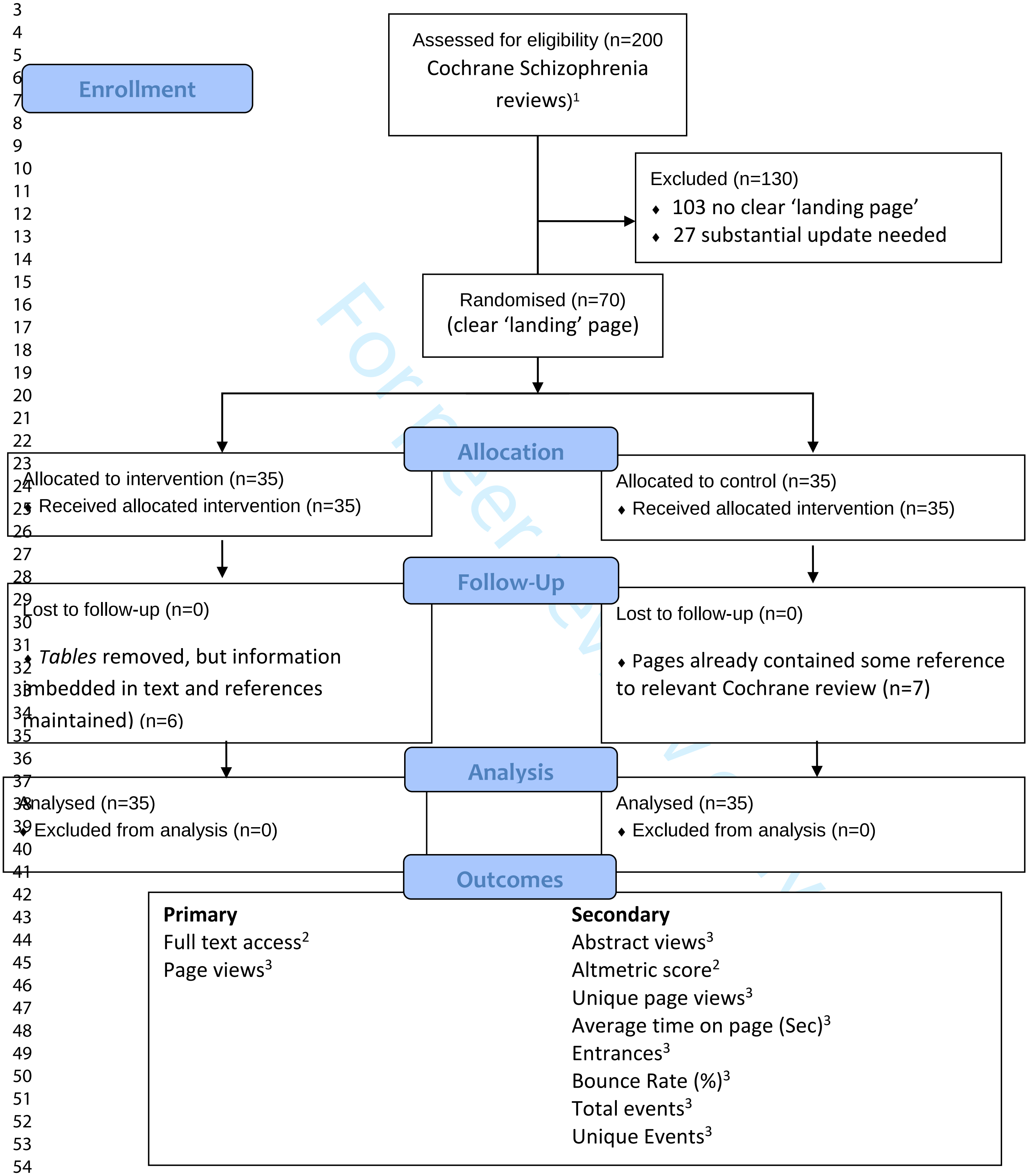

1. 2016 **Data source** 2. John Wiley Ltd 3. Google Analytics

CONSORT checklist of items for reporting pragmatic trials

Section	Item	Standard CONSORT description	Extension for pragmatic trials	Line
Title and abstract	1	How participants were allocated to interventions (eg, "random allocation," "randomised," or "randomly assigned")		CONSORT1
**Introduction**				
Background	2	Scientific background and explanation of rationale	Describe the health or health service problem that the intervention is intended to address and other interventions that may commonly be aimed at this problem	CONSORT2
**Methods**				
Participants	3	Eligibility criteria for participants; settings and locations where the data were collected	Eligibility criteria should be explicitly framed to show the degree to which they include typical participants and/or, where applicable, typical providers (eg, nurses), institutions (eg, hospitals), communities (or localities eg, towns) and settings of care (eg, different healthcare financing systems)	CONSORT3
Interventions	4	Precise details of the interventions intended for each group and how and when they were actually administered	Describe extra resources added to (or resources removed from) usual settings in order to implement intervention. Indicate if efforts were made to standardise the intervention or if the intervention and its delivery were allowed to vary between participants, practitioners, or study sites	CONSORT4

For peer review only

Section	Item	Standard CONSORT description	Extension for pragmatic trials	Line
			Describe the comparator in similar detail to the intervention	
Objectives	5	Specific objectives and hypotheses		CONSORT5
Outcomes	6	Clearly defined primary and secondary outcome measures and, when applicable, any methods used to enhance the quality of measurements (eg, multiple observations, training of assessors)	Explain why the chosen outcomes and, when relevant, the length of follow-up are considered important to those who will use the results of the trial	CONSORT6
Sample size	7	How sample size was determined; explanation of any interim analyses and stopping rules when applicable	If calculated using the smallest difference considered important by the target decision maker audience (the minimally important difference) then report where this difference was obtained	CONSORT7
Randomisation—sequence generation	8	Method used to generate the random allocation sequence, including details of any restriction (eg, blocking, stratification)		CONSORT8
Randomisation—allocation concealment	9	Method used to implement the random allocation sequence (eg, numbered containers or central telephone), clarifying whether the sequence was concealed until interventions were assigned		CONSORT8
Randomisation—implementation	10	Who generated the allocation sequence, who enrolled participants, and who assigned participants to their groups		CONSORT8
Blinding (masking)	11	Whether participants, those administering the interventions, and those assessing the outcomes were blinded to group assignment	If blinding was not done, or was not possible, explain why	CONSORT9

For peer review only

Section	Item	Standard CONSORT description	Extension for pragmatic trials	Line
Statistical methods	12	Statistical methods used to compare groups for primary outcomes; methods for additional analyses, such as subgroup analyses and adjusted analyses		CONSORT10
**Results**				
Participant flow	13	Flow of participants through each stage (a diagram is strongly recommended)—specifically, for each group, report the numbers of participants randomly assigned, receiving intended treatment, completing the study protocol, and analysed for the primary outcome; describe deviations from planned study protocol, together with reasons	The number of participants or units approached to take part in the trial, the number which were eligible, and reasons for non-participation should be reported	Figure 2
Recruitment	14	Dates defining the periods of recruitment and follow-up		CONSORT11 CONSORT12
Baseline data	15	Baseline demographic and clinical characteristics of each group		Table 2
Numbers analysed	16	Number of participants (denominator) in each group included in each analysis and whether analysis was by "intention-to-treat"; state the results in absolute numbers when feasible (eg, 10/20, not 50%)		Figure 2
Outcomes and estimation	17	For each primary and secondary outcome, a summary of results for each group and the estimated effect size and its precision (eg, 95% CI)		Table 3

For peer review only

Section	Item	Standard CONSORT description	Extension for pragmatic trials	Line
Ancillary analyses	18	Address multiplicity by reporting any other analyses performed, including subgroup analyses and adjusted analyses, indicating which are prespecified and which are exploratory		N/A
Adverse events	19	All important adverse events or side effects in each intervention group		N/A
**Discussion**				
Interpretation	20	Interpretation of the results, taking into account study hypotheses, sources of potential bias or imprecision, and the dangers associated with multiplicity of analyses and outcomes		CONSORT13
Generalisability	21	Generalisability (external validity) of the trial findings	Describe key aspects of the setting which determined the trial results. Discuss possible differences in other settings where clinical traditions, health service organisation, staffing, or resources may vary from those of the trial	CONSORT14
Overall evidence	22	General interpretation of the results in the context of current evidence		CONSORT14

Cite as: *Zwarenstein M, Treweek S, Gagnier JJ, Altman DG, Tunis S, Haynes B, Oxman AD, Moher D for the CONSORT and Pragmatic Trials in Healthcare (Practihc) group. Improving the reporting of pragmatic trials: an extension of the CONSORT statement. BMJ 2008; 337;a2390.*

For peer review only
